# Supplementary material for: Prevalence of low birth weight and associated factors in Ethiopia: An umbrella review of systematic review and meta-analyses
Source: PLOS Glob Public Health. 2025 May 8;5(5):e0004556. doi: 10.1371/journal.pgph.0004556 (PMC12061095; doi:10.1371/journal.pgph.0004556)
Supplement: S1 File — (DOCX) [file pgph.0004556.s002.docx]

From PubMed data base (566), Google scholar (223), and Science direct (54) articles using the following search term

(((("infant, low birth weight"[MeSH Terms] AND "Systematic Review"[Publication Type]) OR "Systematic Reviews as Topic"[MeSH Terms] OR "Systematic Review"[All Fields]) AND "meta analysis"[Publication Type]) OR "Meta-Analysis as Topic"[MeSH Terms] OR "meta analysis"[All Fields]) AND "Ethiopia"[MeSH Terms]

From HINARI data base (49) and web of science (26) articles using the following search term

(Low birth weight) AND (systematic review) AND (meta-analysis) AND (Ethiopia)
