# Supplementary material for: Prevalence of low birth weight and associated factors in Ethiopia: An umbrella review of systematic review and meta-analyses
Source: PLOS Glob Public Health. 2025 May 8;5(5):e0004556. doi: 10.1371/journal.pgph.0004556 (PMC12061095; doi:10.1371/journal.pgph.0004556)
Supplement: S1 Table — (DOCX) [file pgph.0004556.s003.docx]

A total of 918 articles yielded from the database search. Of these, 322 articles from the identified studies were removed due to duplication. Subsequently, 578 out of 596 articles were excluded after reviewing the title and abstract. Upon a full-text review of the remaining 18 articles, seven SRMA studies were excluded for various reasons.

| S.no | Article title | Included | Excluded | Reason | URL |
| --- | --- | --- | --- | --- | --- |
| 1 | Efficacy and safety of artemether-lumefantrine for treatment of uncomplicated Plasmodium falciparum malaria in Ethiopia: a systematic review and meta-analysis |  | √ | Excluded based on title and abstract |  |
| 2 | Chronic Malnutrition Among Under Five Children of Ethiopia May Not Be Economic. A Systematic Review and Meta-Analysis |  | √ | Excluded based on title and abstract |  |
| 3 | Non-adherence to self-care and associated factors among diabetes adult population in Ethiopian: A systemic review with meta-analysis |  | √ | Excluded based on title and abstract |  |
| 4 | Level of dietary adherence and determinants among type 2 diabetes population in Ethiopian: A systemic review with meta-analysis |  | √ | Excluded based on title and abstract |  |
| 5 | The burden of stroke and modifiable risk factors in Ethiopia: A systemic review and meta-analysis |  | √ | Excluded based on title and abstract |  |
| 6 | Neurosurgery Research Output in Ethiopia: A Scoping Review |  | √ | Excluded based on title and abstract |  |
| 7 | First trimester antenatal care contact in Africa: a systematic review and meta-analysis of prevalence and contributing factors |  | √ | Excluded based on title and abstract |  |
| 8 | Nutritional Status of Under Five Children in Ethiopia: A Systematic Review and Meta-Analysis |  | √ | Excluded based on title and abstract |  |
| 9 | HIV patients retention and attrition in care and their determinants in Ethiopia: a systematic review and meta-analysis |  | √ | Excluded based on title and abstract |  |
| 10 | Open defecation-free slippage and its associated factors in Ethiopia: a systematic review |  | √ | Excluded based on title and abstract |  |
| 11 | The impact of access to water supply and sanitation on the prevalence of active trachoma in Ethiopia: A systematic review and meta-analysis |  | √ | Excluded based on title and abstract |  |
| 12 | A systematic review and meta-analysis of adolescent nutrition in Ethiopia: Transforming adolescent lives through nutrition (TALENT) initiative |  | √ | Excluded based on title and abstract |  |
| 13 | Estimate the burden of sexual dysfunction due to non-communicable diseases in Ethiopia: Systematic review and meta-analysis |  | √ | Excluded based on title and abstract |  |
| 14 | Antimicrobial prescription patterns in East Africa: a systematic review |  | √ | Excluded based on title and abstract |  |
| 15 | Magnitude and predictors of unfavorable management outcome in surgically treated patients with intestinal obstruction in Ethiopia: a systematic review and meta-analysis |  | √ | Excluded based on title and abstract |  |
| 16 | Non-prescribed drug use and predictors among pregnant women in Ethiopia: systematic review and meta-analysis |  | √ | Excluded based on title and abstract |  |
| 17 | Herbal medicine use and predictors among pregnant women attending antenatal care in Ethiopia: a systematic review and meta-analysis |  | √ | Excluded based on title and abstract |  |
| 18 | Anaemia and its associated factors among diabetes mellitus patients in Ethiopia: A systematic review and meta-analysis |  | √ | Excluded based on title and abstract |  |
| 19 | Knowledge, attitude, and uptake of human papillomavirus vaccine among adolescent schoolgirls in Ethiopia: a systematic review and meta-analysis |  | √ | Excluded based on title and abstract |  |
| 20 | Utilization of non-pneumatic anti-shock garment for treating obstetric hemorrhage and associated factors among obstetric care providers in Ethiopia: A systematic review and meta-analysis |  | √ | Excluded based on title and abstract |  |
| 21 | Vaginal birth after cesarean section and its associated factors in Ethiopia: a systematic review and meta-analysis |  | √ | Excluded based on title and abstract |  |
| 22 | Vaginal birth after cesarean section and its associated factors in Ethiopia: a systematic review and meta-analysis |  | √ | Excluded based on title and abstract |  |
| 23 | Burden of obstructed labor in ethiopia: A systematic review and meta-analysis |  | √ | Excluded based on title and abstract |  |
| 24 | Knowledge and attitude towards Covid-19 vaccine in Ethiopia: Systematic review and meta-analysis |  | √ | Excluded based on title and abstract |  |
| 25 | Adherence to option B+ and its association with disclosure status and counseling among HIV-positive pregnant and lactating women in Ethiopia: systematic review and meta-analysis |  | √ | Excluded based on title and abstract |  |
| 26 | Pregnancy in the Sickle Cell Disease and Fetomaternal Outcomes in Different Sickle cell Genotypes: A Systematic Review and Meta- Analysis |  | √ | Excluded based on title and abstract |  |
| 27 | Availability of adequately iodized salt at the household level in Ethiopia: A systematic review and meta-analysis |  | √ | Excluded based on title and abstract |  |
| 28 | Essential newborn care utilization and associated factors in Ethiopia: a systematic review and meta-analysis |  | √ | Excluded based on title and abstract |  |
| 29 | Magnitude and Predictors of Pneumonia among Under-Five Children in Ethiopia: A Systematic Review and Meta-Analysis |  | √ | Excluded based on title and abstract |  |
| 30 | Metabolic risk factors for non-communicable diseases in Ethiopia: a systematic review and meta-analysis |  | √ | Excluded based on title and abstract |  |
| 31 | Intimate partner violence and associated factors among pregnant women in Ethiopia: a systematic review and meta-analysis |  | √ | Excluded based on title and abstract |  |
| 32 | Prevalence and determinants of diarrhea among under-five children in Ethiopia: A systematic review and meta-analysis |  | √ | Excluded based on title and abstract |  |
| 33 | Prevalence of carbapenemase-producing Enterobacteriaceae from human clinical samples in Ethiopia: a systematic review and meta-analysis |  | √ | Excluded based on title and abstract |  |
| 34 | Prevalence of multidrug-resistant bacteria in Ethiopia: a systematic review and meta-analysis |  | √ | Excluded based on title and abstract |  |
| 35 | Magnitude of Cryptococcosis among HIV patients in sub-Saharan Africa countries: a systematic review and meta-analysis |  | √ | Excluded based on title and abstract |  |
| 36 | Epidemiology of podoconiosis in Ethiopia: a systematic review and meta-analysis protocol |  | √ | Excluded based on title and abstract |  |
| 37 | Prevalence and factors associated with intestinal parasitic infections among food handlers working at higher public University student's cafeterias and public food establishments in Ethiopia: a systematic review and meta-analysis |  | √ | Excluded based on title and abstract |  |
| 38 | Risk factors associated with drug-resistant tuberculosis in Ethiopia: A systematic review and meta-analysis |  | √ | Excluded based on title and abstract |  |
| 39 | Poor treatment outcome and its predictors among drug-resistant tuberculosis patients in Ethiopia: A systematic review and meta-analysis |  | √ | Excluded based on title and abstract |  |
| 40 | Intestinal parasites co-infection among tuberculosis patients in Ethiopia: a systematic review and meta-analysis |  | √ | Excluded based on title and abstract |  |
| 41 | Prevalence and determinants of uterine rupture in Ethiopia: a systematic review and meta-analysis |  | √ | Excluded based on title and abstract |  |
| 42 | Knowledge of preconception care and its association with family planning utilization among women in Ethiopia: meta-analysis |  | √ | Excluded based on title and abstract |  |
| 43 | Hepatitis B Virus Infection and Its Determinants among Pregnant Women in Ethiopia: A Systematic Review and Meta-Analysis |  | √ | Excluded based on title and abstract |  |
| 44 | Healthcare-associated infection and its determinants in Ethiopia: A systematic review and meta-analysis |  | √ | Excluded based on title and abstract |  |
| 45 | Association of age and colostrum discarding with breast-feeding practice in Ethiopia: systematic review and meta-analyses |  | √ | Excluded based on title and abstract |  |
| 46 | Prevalence and associated factors of khat chewing among students in Ethiopia: a protocol for systematic review and meta-analysis |  | √ | Excluded based on title and abstract |  |
| 47 | Internalised stigma among people with mental illness in Africa, pooled effect estimates and subgroup analysis on each domain: systematic review and meta-analysis |  | √ | Excluded based on title and abstract |  |
| 48 | Prevalence of depression in people with tuberculosis in East Africa: a systematic review and meta-analysis |  | √ | Excluded based on title and abstract |  |
| 49 | Impact of multidrug-resistant tuberculosis and its medications on adverse maternal and perinatal outcomes: protocol for a systematic review and meta-analysis |  | √ | Excluded based on title and abstract |  |
| 50 | Treatment Outcomes Among Pregnant Patients With Multidrug-Resistant Tuberculosis: A Systematic Review and Meta-analysis |  | √ | Excluded based on title and abstract |  |
| 51 | Mapping tuberculosis prevalence in Ethiopia using geospatial meta-analysis |  | √ | Excluded based on title and abstract |  |
| 52 | Patient delay in the diagnosis of tuberculosis in Ethiopia: a systematic review and meta-analysis |  | √ | Excluded based on title and abstract |  |
| 53 | Magnitude of risk factors and in-hospital mortality of stroke in Ethiopia: a systematic review and meta-analysis |  | √ | Excluded based on title and abstract |  |
| 54 | Prevalence and determinants of unintended pregnancy in Ethiopia: A systematic review and meta-analysis of observational studies |  | √ | Excluded based on title and abstract |  |
| 55 | Child Wasting in Emergency Pockets: A Meta-Analysis of Small-Scale Surveys from Ethiopia |  | √ | Excluded based on title and abstract |  |
| 56 | Uncontrolled hypertension in Ethiopia: a systematic review and meta-analysis of institution-based observational studies |  | √ | Excluded based on title and abstract |  |
| 57 | Alcohol use and associated factors among high school, college and university students in Ethiopia, systematic review, and meta-analysis, 2018 |  | √ | Excluded based on title and abstract |  |
| 58 | A Systematic Review and Meta-Analysis of Epidemiology of Risky Sexual Behaviors in College and University Students in Ethiopia, 2018 |  | √ | Excluded based on title and abstract |  |
| 59 | The Prevalence of Metabolic Syndrome in Ethiopian Population: A Systematic Review and Meta-analysis |  | √ | Excluded based on title and abstract |  |
| 60 | Undiagnosed hypertension and associated factors among adults in ethiopia: a systematic review and meta-analysis |  | √ | Excluded based on title and abstract |  |
| 61 | Antiseizure medication nonadherence and its associated factors among Epileptic patients in Ethiopia, a systematic review and meta-analysis |  | √ | Excluded based on title and abstract |  |
| 62 | Prevalence of depression among students at Ethiopian universities and associated factors: A systematic review and meta-analysis |  | √ | Excluded based on title and abstract |  |
| 63 | Women's decisions regarding family planning use and its determinants in Ethiopia: A systematic review and meta-analysis protocol |  | √ | Excluded based on title and abstract |  |
| 64 | Preventive practices toward sexually transmitted infections and their determinants among young people in Ethiopia: A protocol for systematic review and meta-analysis |  | √ | Excluded based on title and abstract |  |
| 65 | Male involvement in family planning use and its determinants in Ethiopia: a systematic review and meta-analysis protocol |  | √ | Excluded based on title and abstract |  |
| 66 | Lost to follow-up and associated factors among patients with drug resistant tuberculosis in Ethiopia: A systematic review and meta-analysis |  | √ | Excluded based on title and abstract |  |
| 67 | The prevalence of cardiovascular disease in Ethiopia: a systematic review and meta-analysis of institutional and community-based studies |  | √ | Excluded based on title and abstract |  |
| 68 | Intestinal parasitic infections and associated factors among pregnant women in Ethiopia: a systematic review and meta-analysis |  | √ | Excluded based on title and abstract |  |
| 69 | Prevalence of visual impairment and associated factors among children in Ethiopia: Systematic review and meta-analysis |  | √ | Excluded based on title and abstract |  |
| 70 | Contraceptive dynamics among women with disabilities of reproductive age in Ethiopia: systematic review |  | √ | Excluded based on title and abstract |  |
| 71 | The psychological impact of COVID-19 pandemic and associated factors among college and university students in Ethiopia: a systematic review and meta-analysis, 2022 |  | √ | Excluded based on title and abstract |  |
| 72 | Unknown HIV status and the TB/HIV collaborative control program in Ethiopia: systematic review and meta-analysis |  | √ | Excluded based on title and abstract |  |
| 73 | Gestational weight gain and its effect on birth outcomes in sub-Saharan Africa: Systematic review and meta-analysis |  | √ | Excluded based on title and abstract |  |
| 74 | Infectious and parasitic diseases of poultry in Ethiopia: a systematic review and meta-analysis |  | √ | Excluded based on title and abstract |  |
| 75 | Prevalence of Work-Related Injury and Its Determinants among Construction Workers in Ethiopia: A Systematic Review and Meta-Analysis |  | √ | Excluded based on title and abstract |  |
| 76 | A meta-analysis of contagious caprine pleuropneumonia (CCPP) in Ethiopia |  | √ | Excluded based on title and abstract |  |
| 77 | Major vectors and vector-borne diseases in small ruminants in Ethiopia: A systematic review |  | √ | Excluded based on title and abstract |  |
| 78 | Mange mite infestation in small ruminants in Ethiopia: Systematic review and meta-analysis |  | √ | Excluded based on title and abstract |  |
| 79 | Meta-analysis of Brucella seroprevalence in dairy cattle of Ethiopia |  | √ | Excluded based on title and abstract |  |
| 80 | Gastrointestinal nematode infection in small ruminants in Ethiopia: A systematic review and meta-analysis |  | √ | Excluded based on title and abstract |  |
| 81 | Systematic review and meta-analysis of metacestodes prevalence in small ruminants in Ethiopia |  | √ | Excluded based on title and abstract |  |
| 82 | Tuberculosis in small ruminants and dromedary camels in Ethiopia: A systematic review and meta-analysis |  | √ | Excluded based on title and abstract |  |
| 83 | Lungworm infection in small ruminants in Ethiopia: Systematic review and meta-analysis |  | √ | Excluded based on title and abstract |  |
| 84 | Bovine cysticercosis in Ethiopia: A systematic review and meta-analysis of prevalence from abattoir-based surveys |  | √ | Excluded based on title and abstract |  |
| 85 | Financial burden of tuberculosis diagnosis and treatment for patients in Ethiopia: a systematic review and meta-analysis |  | √ | Excluded based on title and abstract |  |
| 86 | High burden of undernutrition among primary school-aged children and its determinant factors in Ethiopia; a systematic review and meta-analysis |  | √ | Excluded based on title and abstract |  |
| 87 | Prevalence of intestinal parasitic infection and its associated factors among primary school students in Ethiopia: A systematic review and meta-analysis |  | √ | Excluded based on title and abstract |  |
| 88 | Magnitude and causes of first-line antiretroviral therapy regimen changes among HIV patients in Ethiopia: a systematic review and meta-analysis |  | √ | Excluded based on title and abstract |  |
| 89 | Prevalence of visual impairment due to refractive error among children and adolescents in Ethiopia: A systematic review and meta-analysis |  | √ | Excluded based on title and abstract |  |
| 90 | Full-dose hepatitis B virus vaccination coverage and associated factors among health care workers in Ethiopia: A systematic review and meta-analysis |  | √ | Excluded based on title and abstract |  |
| 91 | Therapeutic efficacy of artemether-lumefantrine in the treatment of uncomplicated Plasmodium falciparum malaria in Ethiopia: a systematic review and meta-analysis |  | √ | Excluded based on title and abstract |  |
| 92 | A systematic review and meta-analysis of cardiovascular diseases and associated factors among diabetes mellitus patients in Ethiopia |  | √ | Excluded based on title and abstract |  |
| 93 | A systematic review and meta-analysis of epidemiology of depression in people living with HIV in east Africa |  | √ | Excluded based on title and abstract |  |
| 94 | Prevalence and determinants of antenatal depression in Ethiopia: A systematic review and meta-analysis |  | √ | Excluded based on title and abstract |  |
| 95 | Epidemiology of khat (Catha edulis) consumption among university students: a meta-analysis |  | √ | Excluded based on title and abstract |  |
| 96 | The epidemiology of alcohol consumption in Ethiopia: a systematic review and meta-analysis |  | √ | Excluded based on title and abstract |  |
| 97 | A systematic review and meta-analysis protocol on stunting and its determinants among school-age children (6-14years) in Ethiopia |  | √ | Excluded based on title and abstract |  |
| 98 | Knowledge and utilisation of preconception care and associated factors among women in Ethiopia: systematic review and meta-analysis |  | √ | Excluded based on title and abstract |  |
| 99 | Knowledge and practice of essential newborn care and associated factors among women in Ethiopia: systematic review and meta-analysis |  | √ | Excluded based on title and abstract |  |
| 100 | Dietary diversity practice and associated factors among adolescents in Ethiopia, systematic review and meta-analysis |  | √ | Excluded based on title and abstract |  |
| 101 | Incidence, causes, and maternofetal outcomes of obstructed labor in Ethiopia: systematic review and meta-analysis |  | √ | Excluded based on title and abstract |  |
| 102 | Partograph utilization as a decision-making tool and associated factors among obstetric care providers in Ethiopia: a systematic review and meta-analysis |  | √ | Excluded based on title and abstract |  |
| 103 | Prevalence of potential drug-drug interactions and associated factors among outpatients and inpatients in Ethiopian hospitals: a systematic review and meta-analysis of observational studies |  | √ | Excluded based on title and abstract |  |
| 104 | Determinants of diabetic nephropathy among diabetic patients in Ethiopia: Systematic review and meta-analysis |  | √ | Excluded based on title and abstract |  |
| 105 | Determinants of diabetic retinopathy in Ethiopia: A systematic review and meta-analysis |  | √ | Excluded based on title and abstract |  |
| 106 | Prevalence and associated factors of scabies in Ethiopia: systematic review and Meta-analysis |  | √ | Excluded based on title and abstract |  |
| 107 | Prevalence of anemia and its associated factors among children aged 6-23 months, in Ethiopia: a systematic review and meta analysis |  | √ | Excluded based on title and abstract |  |
| 108 | The state of birth asphyxia in Ethiopia: An umbrella review of systematic review and meta-analysis reports, 2020 |  | √ | Excluded based on title and abstract |  |
| 109 | Willingness to pay for social health insurance in Ethiopia: A systematic review and meta-analysis |  | √ | Excluded based on title and abstract |  |
| 110 | The impact of community-based health insurance on universal health coverage in Ethiopia: a systematic review and meta-analysis |  | √ | Excluded based on title and abstract |  |
| 111 | Global burden of potentially life-threatening maternal conditions: a systematic review and meta-analysis |  | √ | Excluded based on title and abstract |  |
| 112 | Production, reproduction and some adaptation characteristics of Boran cattle breed under changing climate: A systematic review and meta-analysis |  | √ | Excluded based on title and abstract |  |
| 113 | Adherence to self-care practices and associated factors among heart failure patients in Ethiopia: A systematic review and meta-analysis |  | √ | Excluded based on title and abstract |  |
| 114 | Burden and predictors of heart failure treatment outcomes in Ethiopia: A systematic review and meta-analysis protocol |  | √ | Excluded based on title and abstract |  |
| 115 | Prevalence of Group B Streptococcus Recto-Vaginal Colonization, Vertical Transmission, and Antibiotic Susceptibility Among Pregnant Women in Ethiopia: A Systematic Review and Meta-Analysis |  | √ | Excluded based on title and abstract |  |
| 116 | Under-five anemia and its associated factors with dietary diversity, food security, stunted, and deworming in Ethiopia: systematic review and meta-analysis |  | √ | Excluded based on title and abstract |  |
| 117 | Neonatal mortality and its association with antenatal care visits among live births in Ethiopia: a systematic review and meta-analysis |  | √ | Excluded based on title and abstract |  |
| 118 | Macrosomia and its predictors in pregnant women with diabetes in Ethiopia |  | √ | Excluded based on title and abstract |  |
| 119 | Late presentation of HIV positive adults and its predictors to HIV/AIDS care in Ethiopia: a systematic review and meta-analysis |  | √ | Excluded based on title and abstract |  |
| 120 | Pediatrics HIV-positive status disclosure and its predictors in Ethiopia: a systematic review and meta-analysis |  | √ | Excluded based on title and abstract |  |
| 121 | Magnitude and predictors of common mental disorder among people with HIV/AIDS in Ethiopia: a systematic review and meta-analysis |  | √ | Excluded based on title and abstract |  |
| 122 | The prevalence of vancomycin-resistant Staphylococcus aureus in Ethiopia: a systematic review and meta-analysis |  | √ | Excluded based on title and abstract |  |
| 123 | Prevalence of neonatal hypothermia and its associated factors in East Africa: a systematic review and meta-analysis |  | √ | Excluded based on title and abstract |  |
| 124 | Hepatitis viruses in Ethiopia: a systematic review and meta-analysis |  | √ | Excluded based on title and abstract |  |
| 125 | Meta-analyses of fertility desires of people living with HIV |  | √ | Excluded based on title and abstract |  |
| 126 | Prevalence of hypertensive disorders of pregnancy in Ethiopia: a systemic review and meta-analysis |  | √ | Excluded based on title and abstract |  |
| 127 | Birth preparedness and complication readiness among pregnant women in Ethiopia: a systematic review and Meta-analysis |  | √ | Excluded based on title and abstract |  |
| 128 | Prevalence and associated factors of zinc deficiency among pregnant women and children in Ethiopia: a systematic review and meta-analysis |  | √ | Excluded based on title and abstract |  |
| 129 | Prevalence and drug resistance patterns of Gram-negative enteric bacterial pathogens from diarrheic patients in Ethiopia: A systematic review and meta-analysis |  | √ | Excluded based on title and abstract |  |
| 130 | Gestational diabetes mellitus and its associated factors in Ethiopia: a systematic review and meta-analysis |  | √ | Excluded based on title and abstract |  |
| 131 | Utilization and its factors of post abortion modern contraceptive in Ethiopia: a systematic review and meta-analysis |  | √ | Excluded based on title and abstract |  |
| 132 | Domestic violence related disclosure among women and girls in Ethiopia: a systematic review and meta-analysis |  | √ | Excluded based on title and abstract |  |
| 133 | Prevalence of suicidal ideation, suicidal attempt and completed suicide in Ethiopia: a systematic review and meta-analysis protocol |  | √ | Excluded based on title and abstract |  |
| 134 | Determinants of Intimate Partner Violence against Pregnant Women in Ethiopia: A Systematic Review and Meta-Analysis |  | √ | Excluded based on title and abstract |  |
| 135 | Unfavorable public attitude toward people with epilepsy in Ethiopia: A systematic review and meta-analysis study |  | √ | Excluded based on title and abstract |  |
| 136 | National estimate and associated factors of myopia among schoolchildren in Ethiopia: A systematic review and meta-analysis |  | √ | Excluded based on title and abstract |  |
| 137 | Magnitude and associated factors of urinary tract infections among adults living with HIV in Ethiopia. Systematic review and meta-analysis |  | √ | Excluded based on title and abstract |  |
| 138 | Tinea capitis among schoolchildren in Ethiopia: A systematic review and meta analysis |  | √ | Excluded based on title and abstract |  |
| 139 | Malaria among under-five children in Ethiopia: a systematic review and meta-analysis |  | √ | Excluded based on title and abstract |  |
| 140 | Full immunization coverage and associated factors among children age 12-23 months in Ethiopia: systematic review and meta-analysis of observational studies |  | √ | Excluded based on title and abstract |  |
| 141 | The prevalence of thrombocytopenia and leucopenia among people living with HIV/AIDS in Ethiopia: A systematic review and meta-analysis |  | √ | Excluded based on title and abstract |  |
| 142 | Prevalence of Glycemic Control and Factors Associated With Poor Glycemic Control: A Systematic Review and Meta-analysis |  | √ | Excluded based on title and abstract |  |
| 143 | Treatment outcomes of severe acute malnutrition and predictors of recovery in under-five children treated within outpatient therapeutic programs in Ethiopia: a systematic review and meta-analysis |  | √ | Excluded based on title and abstract |  |
| 144 | Atovaquone-proguanil for treating uncomplicated Plasmodium falciparum malaria |  | √ | Excluded based on title and abstract |  |
| 145 | Comorbidity of Geo-Helminthes among Malaria Outpatients of the Health Facilities in Ethiopia: Systematic Review and Meta-Analysis |  | √ | Excluded based on title and abstract |  |
| 146 | The burden of household out-of-pocket healthcare expenditures in Ethiopia: a systematic review and meta-analysis |  | √ | Excluded based on title and abstract |  |
| 147 | Prevalence and associated factors of self-medication in worldwide pregnant women: systematic review and meta-analysis |  | √ | Excluded based on title and abstract |  |
| 148 | Highlights from this issue |  | √ | Excluded based on title and abstract |  |
| 149 | Utilization and Determinants of Postnatal Care Services in Ethiopia: A Systematic Review and Meta-Analysis |  | √ | Excluded based on title and abstract |  |
| 150 | Bacterial profile and antimicrobial resistance patterns of common bacteria among pregnant women with bacteriuria in Ethiopia: a systematic review and meta-analysis |  | √ | Excluded based on title and abstract |  |
| 151 | Epidemiology of intestinal parasitic infections in preschool and school-aged Ethiopian children: a systematic review and meta-analysis |  | √ | Excluded based on title and abstract |  |
| 152 | Prevalence of soil-transmitted helminths infections among preschool and school-age children in Ethiopia: a systematic review and meta-analysis |  | √ | Excluded based on title and abstract |  |
| 153 | Epidemiology of staphylococci species and their antimicrobial-resistance among patients with wound infection in Ethiopia: a systematic review and meta-analysis |  | √ | Excluded based on title and abstract |  |
| 154 | Burden of intestinal parasitic infections among pregnant women in Ethiopia: a systematic review and meta-analysis |  | √ | Excluded based on title and abstract |  |
| 155 | Weight loss and mortality in people living with HIV: a systematic review and meta-analysis |  | √ | Excluded based on title and abstract |  |
| 156 | Balantidiasis in humans: A systematic review and meta-analysis |  | √ | Excluded based on title and abstract |  |
| 157 | Prevalence of occupational respiratory symptoms and associated factors among industry workers in Ethiopia: A systematic review and meta-analysis |  | √ | Excluded based on title and abstract |  |
| 158 | Prevalence of mental distress and associated factors among university students in Ethiopia: a meta-analysis |  | √ | Excluded based on title and abstract |  |
| 159 | Prevalence and determinants of mental distress among university students in Ethiopia: a systematic review protocol |  | √ | Excluded based on title and abstract |  |
| 160 | A systematic review and meta-analysis of the effect of short birth interval on infant mortality in Ethiopia |  | √ | Excluded based on title and abstract |  |
| 161 | Effect of perinatal depression on birth and infant health outcomes: a systematic review and meta-analysis of observational studies from Africa |  | √ | Excluded based on title and abstract |  |
| 162 | Role of maternity waiting homes in the reduction of maternal death and stillbirth in developing countries and its contribution for maternal death reduction in Ethiopia: a systematic review and meta-analysis |  | √ | Excluded based on title and abstract |  |
| 163 | Epidemiology of bovine schistosomiasis and associated risk factors in Ethiopia: A systematic review with meta-analysis of published articles, 2008-2018 |  | √ | Excluded based on title and abstract |  |
| 164 | A systematic review and meta-analysis on adoption of WHO-recommended infant feeding practices among HIV positive mothers in Ethiopia |  | √ | Excluded based on title and abstract |  |
| 165 | Hypertriglyceridemia and Other Plasma Lipid Profile Abnormalities among People Living with Diabetes Mellitus in Ethiopia: A Systematic Review and Meta-Analysis |  | √ | Excluded based on title and abstract |  |
| 166 | Prevalence and Genetic Diversity of Rotaviruses among under-Five Children in Ethiopia: A Systematic Review and Meta-Analysis |  | √ | Excluded based on title and abstract |  |
| 167 | Pre-marital sex and its association with peer pressure and watching pornography among young individuals in Ethiopia: a systematic review and meta-analysis |  | √ | Excluded based on title and abstract |  |
| 168 | Short birth spacing and its association with maternal educational status, contraceptive use, and duration of breastfeeding in Ethiopia. A systematic review and meta-analysis |  | √ | Excluded based on title and abstract |  |
| 169 | HIV risk behavior and associated factors among people living with HIV/AIDS in Ethiopia: A systematic review and meta-analysis |  | √ | Excluded based on title and abstract |  |
| 170 | The effect of lack of ANC visit and unwanted pregnancy on home child-birth in Ethiopia: a systematic review and meta-analysis |  | √ | Excluded based on title and abstract |  |
| 171 | Toxoplasmosis infection among pregnant women in Africa: A systematic review and meta-analysis |  | √ | Excluded based on title and abstract |  |
| 172 | Food taboo practices and associated factors among pregnant women in Ethiopia: a systematic review and meta-analysis |  | √ | Excluded based on title and abstract |  |
| 173 | Drought and child mortality: a meta-analysis of small-scale surveys from Ethiopia |  | √ | Excluded based on title and abstract |  |
| 174 | Drought, conflict and children's undernutrition in Ethiopia 2000-2013: a meta-analysis |  | √ | Excluded based on title and abstract |  |
| 175 | The prevalence of stroke among adults in Ethiopia from 2012 to 2022: A systematic review and meta-analysis protocol |  | √ | Excluded based on title and abstract |  |
| 176 | Fluoride concentration in ground water and prevalence of dental fluorosis in Ethiopian Rift Valley: systematic review and meta-analysis |  | √ | Excluded based on title and abstract |  |
| 177 | Women's knowledge towards neonatal danger signs and its associated factors in Ethiopia: a systematic review and meta-analysis |  | √ | Excluded based on title and abstract |  |
| 178 | Women's satisfaction with existing labour and delivery services in Ethiopia: a systematic review and meta-analysis |  | √ | Excluded based on title and abstract |  |
| 179 | Utilization of family planning and associated factors among women with disabilities in ethiopia: A systematic review and meta-analysis |  | √ | Excluded based on title and abstract |  |
| 180 | Prevalence and risk factors for road traffic injuries and mortalities in Ethiopia: systematic review and meta-analysis |  | √ | Excluded based on title and abstract |  |
| 181 | Seroprevalence of Hepatitis C Viral Infection in Ethiopia: A Systematic Review and Meta-Analysis |  | √ | Excluded based on title and abstract |  |
| 182 | Prevalence and determinants of neonatal near miss in Ethiopia: A systematic review and meta-analysis |  | √ | Excluded based on title and abstract |  |
| 183 | Spatial distribution of podoconiosis in relation to environmental factors in Ethiopia: a historical review |  | √ | Excluded based on title and abstract |  |
| 184 | Determinants of birth asphyxia among newborns in Ethiopia: A systematic review and meta-analysis |  | √ | Excluded based on title and abstract |  |
| 185 | Prevalence of goiter among children in Ethiopia and associated factors: a systematic review and meta-analysis |  | √ | Excluded based on title and abstract |  |
| 186 | The effect of disclosure on adherence to antiretroviral therapy among adults living with HIV in Ethiopia: a systematic review and meta-analysis |  | √ | Excluded based on title and abstract |  |
| 187 | Effect of Preterm Birth on the Risk of Adverse Perinatal and Neonatal Outcomes in Ethiopia: A Systematic Review and Meta-Analysis |  | √ | Excluded based on title and abstract |  |
| 188 | Prevalence and predictors of uterine rupture among Ethiopian women: A systematic review and meta-analysis |  | √ | Excluded based on title and abstract |  |
| 189 | Cervical cancer screening utilization and predictors among eligible women in Ethiopia: A systematic review and meta-analysis |  | √ | Excluded based on title and abstract |  |
| 190 | Maternal and perinatal mortality and morbidity of uterine rupture and its association with prolonged duration of operation in Ethiopia: A systematic review and meta-analysis |  | √ | Excluded based on title and abstract |  |
| 191 | Adherence of iron and folic acid supplementation and determinants among pregnant women in Ethiopia: a systematic review and meta-analysis |  | √ | Excluded based on title and abstract |  |
| 192 | Postpartum depression and its association with intimate partner violence and inadequate social support in Ethiopia: a systematic review and meta-analysis |  | √ | Excluded based on title and abstract |  |
| 193 | Household water treatment practice and associated factors in Ethiopia: A systematic review and meta-analysis |  | √ | Excluded based on title and abstract |  |
| 194 | Food safety knowledge, attitude, and practice of street food vendors and associated factors in low-and middle-income countries: A Systematic review and Meta-analysis |  | √ | Excluded based on title and abstract |  |
| 195 | Body mass index and percent body fat: a meta analysis among different ethnic groups |  | √ | Excluded based on title and abstract |  |
| 196 | Prevalence of episiotomy practice and factors associated with it in Ethiopia, systematic review and meta-analysis |  | √ | Excluded based on title and abstract |  |
| 197 | Prevalence and antimicrobial resistance of coagulase negative staphylococci clinical isolates from Ethiopia: a meta-analysis |  | √ | Excluded based on title and abstract |  |
| 198 | Association between social supports and depression among patients with diabetes mellitus in Ethiopia: a systematic review and meta-analysis |  | √ | Excluded based on title and abstract |  |
| 199 | Bacteriologically confirmed extrapulmonary tuberculosis and the associated risk factors among extrapulmonary tuberculosis suspected patients in Ethiopia: A systematic review and meta-analysis |  | √ | Excluded based on title and abstract |  |
| 200 | Drug resistance and its risk factors among extrapulmonary tuberculosis in Ethiopia: A systematic review and meta-analysis |  | √ | Excluded based on title and abstract |  |
| 201 | Prevalence and associated factor of Campylobacter species among less than 5-year-old children in Ethiopia: a systematic review and meta-analysis |  | √ | Excluded based on title and abstract |  |
| 202 | The prevalence of Listeria species in different food items of animal and plant origin in Ethiopia: a systematic review and meta-analysis |  | √ | Excluded based on title and abstract |  |
| 203 | The Prevalence of Alcohol Consumption Among Pregnant Women in Ethiopia: A Systematic Review and Meta-Analysis |  | √ | Excluded based on title and abstract |  |
| 204 | The prevalence of premenstrual dysphoric disorder among adolescents in Ethiopia: a systematic review and meta-analysis |  | √ | Excluded based on title and abstract |  |
| 205 | The epidemiology of postnatal depression in Ethiopia: a systematic review and meta-analysis |  | √ | Excluded based on title and abstract |  |
| 206 | Prevalence of depression among women with obstetric fistula in low-income African countries: a systematic review and meta-analysis |  | √ | Excluded based on title and abstract |  |
| 207 | Exploring the association between khat use and psychiatric symptoms: a systematic review |  | √ | Excluded based on title and abstract |  |
| 208 | HIV infection and unknown HIV status among tuberculosis patients in Ethiopia: a systematic review and meta-analysis |  | √ | Excluded based on title and abstract |  |
| 209 | Disclosure of HIV seropositivity to sexual partner in Ethiopia: A systematic review |  | √ | Excluded based on title and abstract |  |
| 210 | A systematic review and meta-analysis of vertical transmission route of HIV in Ethiopia |  | √ | Excluded based on title and abstract |  |
| 211 | HIV test coverage among pregnant women in Ethiopia: A systematic review and meta-analysis |  | √ | Excluded based on title and abstract |  |
| 212 | HIV/AIDS treatment failure and associated factors in Ethiopia: meta-analysis |  | √ | Excluded based on title and abstract |  |
| 213 | Adherence to Highly Active Antiretroviral Therapy Among Children in Ethiopia: A Systematic Review and Meta-analysis |  | √ | Excluded based on title and abstract |  |
| 214 | Menstrual abnormality, maternal illiteracy, and household factors as main predictors of anemia among adolescent girls in Ethiopia: Systematic review and meta-analysis |  | √ | Excluded based on title and abstract |  |
| 215 | Epidemiology of depressive disorders in people living with hypertension in Africa: a systematic review and meta-analysis |  | √ | Excluded based on title and abstract |  |
| 216 | Effect of antenatal care on low birth weight: a systematic review and meta-analysis in Africa, 2022 |  | √ | Excluded based on title and abstract |  |
| 217 | Biomass fuel use and acute respiratory infection among children younger than 5 years in Ethiopia: a systematic review and meta-analysis |  | √ | Excluded based on title and abstract |  |
| 218 | Management of neonatal jaundice in low-income and middle-income countries |  | √ | Excluded based on title and abstract |  |
| 219 | Adolescent-Parent Communication on Sexual and Reproductive Health issues in Ethiopia: A Systematic Review and Meta-analysis |  | √ | Excluded based on title and abstract |  |
| 220 | Immunization coverage and its determinant factors among children aged 12-23 months in Ethiopia: a systematic review, and Meta- analysis of cross-sectional studies |  | √ | Excluded based on title and abstract |  |
| 221 | Current treatment of multidrug resistant tuberculosis in Ethiopia: an aggregated and individual patients' data analysis for outcome and effectiveness of the current regimens |  | √ | Excluded based on title and abstract |  |
| 222 | Tuberculosis treatment outcomes in Ethiopia from 2003 to 2016, and impact of HIV co-infection and prior drug exposure: A systematic review and meta-analysis |  | √ | Excluded based on title and abstract |  |
| 223 | Multidrug resistant tuberculosis in Ethiopian settings and its association with previous history of anti-tuberculosis treatment: a systematic review and meta-analysis |  | √ | Excluded based on title and abstract |  |
| 224 | Methicillin resistant Staphylococcus aureus in Ethiopia: a meta-analysis |  | √ | Excluded based on title and abstract |  |
| 225 | Is intimate partner violence and obstetrics characteristics of pregnant women associated with preterm birth in Ethiopia? Umbrella review on preterm birth |  | √ | Excluded based on title and abstract |  |
| 226 | Early newborn bathing practice and its determinants among postpartum women in Ethiopia: a systematic review and meta-analysis |  | √ | Excluded based on title and abstract |  |
| 227 | Vitamin A deficiency among pregnant women in Ethiopia: a systematic review and meta-analysis |  | √ | Excluded based on title and abstract |  |
| 228 | Neonatal sepsis and its predictors in Ethiopia: umbrella reviews of a systematic review and meta-analysis, 2023 |  | √ | Excluded based on title and abstract |  |
| 229 | Antenatal depression and its association with adverse birth outcomes in low and middle-income countries: A systematic review and meta-analysis |  | √ | Excluded based on title and abstract |  |
| 230 | The effect of antenatal care on use of institutional delivery service and postnatal care in Ethiopia: a systematic review and meta-analysis |  | √ | Excluded based on title and abstract |  |
| 231 | Effectiveness of eradication therapy for Helicobacter pylori infection in Africa: a systematic review and meta-analysis |  | √ | Excluded based on title and abstract |  |
| 232 | Performance of rapid diagnostic tests, microscopy, loop-mediated isothermal amplification (LAMP) and PCR for malaria diagnosis in Ethiopia: a systematic review and meta-analysis |  | √ | Excluded based on title and abstract |  |
| 233 | Trichomonas vaginalis infection in Ethiopia: A systematic review and meta-analysis |  | √ | Excluded based on title and abstract |  |
| 234 | The burden of visual impairment among Ethiopian adult population: Systematic review and meta-analysis |  | √ | Excluded based on title and abstract |  |
| 235 | Effect of adverse perinatal outcomes on postpartum maternal mental health in low-income and middle-income countries: a protocol for systematic review |  | √ | Excluded based on title and abstract |  |
| 236 | Prevalence of homebirth preference and associated factors among pregnant women in Ethiopia: Systematic review and meta-analysis |  | √ | Excluded based on title and abstract |  |
| 237 | What determines client satisfaction on labor and delivery service in Ethiopia? systematic review and meta-analysis |  | √ | Excluded based on title and abstract |  |
| 238 | Association between adherence to Antiretroviral Therapy and place of residence among adult HIV infected patients in Ethiopia: A systematic review and meta-analysis |  | √ | Excluded based on title and abstract |  |
| 239 | Stigma and associated factors among people with epilepsy in Ethiopia: A systematic review and meta-analysis |  | √ | Excluded based on title and abstract |  |
| 240 | Seroprevalence and factors associated with hepatitis B virus infection in blood donors in Ethiopia: a systematic review and meta-analysis |  | √ | Excluded based on title and abstract |  |
| 241 | Diabetic retinopathy in Ethiopia: A systematic review and meta-analysis |  | √ | Excluded based on title and abstract |  |
| 242 | Health care seeking behavior among presumptive tuberculosis patients in Ethiopia: a systematic review and meta-analysis |  | √ | Excluded based on title and abstract |  |
| 243 | Intention to use maternal waiting home and its predictors among pregnant women in Ethiopia: systematic review and meta-analysis |  | √ | Excluded based on title and abstract |  |
| 244 | The magnitude of episiotomy among women who gave birth in Ethiopia: Systematic review and meta-analysis |  | √ | Excluded based on title and abstract |  |
| 245 | Knowledge, attitude and practice towards kangaroo mother care among postnatal women in Ethiopia: Systematic review and meta-analysis |  | √ | Excluded based on title and abstract |  |
| 246 | Early marriage and its associated factors among women in Ethiopia: Systematic reviews and meta-analysis |  | √ | Excluded based on title and abstract |  |
| 247 | Global prevalence of post-abortion depression: systematic review and Meta-analysis |  | √ | Excuded based on title and abstract |  |
| 248 | Discontinuation of long acting reversible contraceptive use and its determinants among women in Ethiopia: Systematic review and meta-analysis |  | √ | Excluded based on title and abstract |  |
| 249 | The effect of maternal educational status, antenatal care and resumption of menses on postpartum contraceptive use in Ethiopia: systematic review and meta-analysis |  | √ | Excluded based on title and abstract |  |
| 250 | Prevalence of hospital-acquired infections (HAIs) and associated factors in Ethiopia: a systematic review and meta-analysis protocol |  | √ | Excluded based on title and abstract |  |
| 251 | Adherence to COVID-19 preventive measures and associated factors in Ethiopia: A systematic review and meta-analysis |  | √ | Excluded based on title and abstract |  |
| 252 | Turnover intention among healthcare workers in Ethiopia: a systematic review and meta-analysis |  | √ | Excluded based on title and abstract |  |
| 253 | A meta-analysis of the prevalence of Toxoplasma gondii in animals and humans in Ethiopia |  | √ | Excluded based on title and abstract |  |
| 254 | Antimicrobial resistance pattern of Klebsiella isolated from various clinical samples in Ethiopia: a systematic review and meta-analysis |  | √ | Excluded based on title and abstract |  |
| 255 | Folate deficiency among women of reproductive age in Ethiopia: A systematic review and meta-analysis |  | √ | Excluded based on title and abstract |  |
| 256 | The prevalence and risk factors of work-related musculoskeletal disorders among adults in Ethiopia: a study protocol for extending a systematic review with meta-analysis of observational studies |  | √ | Excluded based on title and abstract |  |
| 257 | Anti-malarial treatment outcomes in Ethiopia: a systematic review and meta-analysis |  | √ | Excluded based on title and abstract |  |
| 258 | Glycemic control among diabetic patients in Ethiopia: A systematic review and meta-analysis |  | √ | Excluded based on title and abstract |  |
| 259 | A systematic review and meta-analysis of the prevalence and predictors of anemia among children in Ethiopia |  | √ | Excluded based on title and abstract |  |
| 260 | Prevalence and predictors of khat chewing among Ethiopian university students: A systematic review and meta-analysis |  | √ | Excluded based on title and abstract |  |
| 261 | Prevalence and associated factors of active trachoma among children in Ethiopia: a systematic review and meta-analysis |  | √ | Excluded based on title and abstract |  |
| 262 | Do pregnant African women exercise? A meta-analysis |  | √ | Excluded based on title and abstract |  |
| 263 | Burden of pelvic organ prolapse in Ethiopia: a systematic review and meta-analysis |  | √ | Excluded based on title and abstract |  |
| 264 | Contraceptive Use and Method Preferences among HIV Positive Women in Ethiopia: A Systematic Review and Meta-analysis |  | √ | Excluded based on title and abstract |  |
| 265 | Prevalence and Associated Factors of Iron Deficiency and Iron Deficiency Anemia Among Under-5 Children: A Systematic Review and Meta-Analysis |  | √ | Excluded based on title and abstract |  |
| 266 | Prevalence and associated factors of treatment failure among children on ART in Ethiopia: A systematic review and meta-analysis |  | √ | Excluded based on title and abstract |  |
| 267 | WOMEN's Knowledge of Obstetric Danger signs in Ethiopia (WOMEN's KODE):a systematic review and meta-analysis |  | √ | Excluded based on title and abstract |  |
| 268 | The protective effect of isoniazid preventive therapy on tuberculosis incidence among HIV positive patients receiving ART in Ethiopian settings: a meta-analysis |  | √ | Excluded based on title and abstract |  |
| 269 | Tuberculosis and its association with CD4(+) T cell count among adult HIV positive patients in Ethiopian settings: a systematic review and meta-analysis |  | √ | Excluded based on title and abstract |  |
| 270 | Seroprevalence of HIV among pregnant women in Ethiopia: a systematic review and meta-analysis |  | √ | Excluded based on title and abstract |  |
| 271 | Sero-prevalence of syphilis and associated factors among pregnant women in Ethiopia: a systematic review and meta-analysis |  | √ | Excluded based on title and abstract |  |
| 272 | Discontinuation from Antiretroviral Therapy: A Continuing Challenge among Adults in HIV Care in Ethiopia: A Systematic Review and Meta-Analysis |  | √ | Excluded based on title and abstract |  |
| 273 | Prevalence and associated factors of client satisfaction with family planning service among family planning users in Ethiopia: a systematic review and meta-analysis |  | √ | Excluded based on title and abstract |  |
| 274 | Prevalence and predictors of anemia among pregnant women in Ethiopia: Systematic review and meta-analysis |  | √ | Excluded based on title and abstract |  |
| 275 | Prevalence and associated factors of premenstrual syndrome among women of the reproductive age group in Ethiopia: Systematic review and meta-analysis |  | √ | Excluded based on title and abstract |  |
| 276 | The burdens, associated factors, and reasons for traditional uvulectomy in Ethiopia: A systematic review and meta-analysis |  | √ | Excluded based on title and abstract |  |
| 277 | Meta-analysis of the prevalence of mastitis and associated risk factors in dairy cattle in Ethiopia |  | √ | Excluded based on title and abstract |  |
| 278 | Prevalence of surgical site infection and its associated factors after cesarean section in Ethiopia: systematic review and meta-analysis |  | √ | Excluded based on title and abstract |  |
| 279 | Impact of cesarean section on timely initiation of breastfeeding in Ethiopia: a systematic review and meta-analysis |  | √ | Excluded based on title and abstract |  |
| 280 | Prevalence of Urinary Tract Infection and Its Associated Factors among Pregnant Women in Ethiopia: A Systematic Review and Meta-Analysis |  | √ | Excluded based on title and abstract |  |
| 281 | Thrombocytopenia among pregnant women in Africa: a systematic review and meta-analysis |  | √ | Excluded based on title and abstract |  |
| 282 | Blood Donation Practice and Associated Factors in Ethiopia: A Systematic Review and Meta-analysis |  | √ | Excluded based on title and abstract |  |
| 283 | Knowledge of blood donation and associated factors in Ethiopia: a systematic review and meta-analysis |  | √ | Excluded based on title and abstract |  |
| 284 | Knowledge on Palliative Care and Associated Factors among Nurses in Ethiopia: A Systematic Review and Meta-Analysis |  | √ | Excluded based on title and abstract |  |
| 285 | Prevalence and Risk Factors for Antenatal Depression in Ethiopia: Systematic Review |  | √ | Excluded based on title and abstract |  |
| 286 | Double burden of malnutrition and associated factors among adolescent in Ethiopia: A systematic review and meta-analysis |  | √ | Excluded based on title and abstract |  |
| 287 | Prevalence and Associated Risk Factors of Intestinal Parasites and Enteric Bacterial Infections among Selected Region Food Handlers of Ethiopia during 2014-2022: A Systematic Review and Meta-Analysis |  | √ | Excluded based on title and abstract |  |
| 288 | Magnitude, Associated Risk Factors, and Trend Comparisons of Urinary Tract Infection among Pregnant Women and Diabetic Patients: A Systematic Review and Meta-Analysis |  | √ | Excluded based on title and abstract |  |
| 289 | Magnitude and Determinants of Intestinal Parasites among Children under Five in Ethiopia During 2010-2023: A Systematic Review and Meta-Analysis |  | √ | Excluded based on title and abstract |  |
| 290 | Occupational stress and associated factors among health care professionals in Ethiopia: a systematic review and meta-analysis |  | √ | Excluded based on title and abstract |  |
| 291 | Under-Nutrition and Associated Factors Among Lactating Mothers in Ethiopia: A Systematic Review and Meta-analysis |  | √ | Excluded based on title and abstract |  |
| 292 | Public knowledge toward Epilepsy and its determinants in Ethiopia: A systematic review and meta-analysis |  | √ | Excluded based on title and abstract |  |
| 293 | Comparison of antibiotic regimens for treating louse-borne relapsing fever: a meta-analysis |  | √ | Excluded based on title and abstract |  |
| 294 | Women's attitude and reasons toward justifying domestic violence in Ethiopia: a systematic review and meta-analysis |  | √ | Excluded based on title and abstract |  |
| 295 | Prevalence of Poor Diabetes Self-Management Behaviors among Ethiopian Diabetes Mellitus Patients: A Systematic Review and Meta-Analysis |  | √ | Excluded based on title and abstract |  |
| 296 | Towards universal health coverage: The level and determinants of enrollment in the Community-Based Health Insurance (CBHI) scheme in Ethiopia: A systematic review and meta-analysis |  | √ | Excluded based on title and abstract |  |
| 297 | The prevalence of Implanon discontinuation and associated factors among Ethiopian women: A systematic review and meta-analysis |  | √ | Excluded based on title and abstract |  |
| 298 | The prevalence of respectful maternity care during childbirth and its determinants in Ethiopia: A systematic review and meta-analysis |  | √ | Excluded based on title and abstract |  |
| 299 | The effect of unemployment and post-natal care on the exclusive breast-feeding practice of women in Ethiopia: a systematic review and meta-analysis |  | √ | Excluded based on title and abstract |  |
| 301 | Ethiopian women's breast cancer self-examination practices and associated factors. A systematic review and meta-analysis |  | √ | Excluded based on title and abstract |  |
| 302 | Prevalence and associated factors of anemia among adolescent girls in Ethiopia: A systematic review and meta-analysis |  | √ | Excluded based on title and abstract |  |
| 303 | Biomedical and public health reviews and meta-analyses in Ethiopia had poor methodological quality: overview of evidence from 1970 to 2018 |  | √ | Excluded based on title and abstract |  |
| 304 | Sociodemographic Factors Predicting Exclusive Breastfeeding in Ethiopia: Evidence from a Meta-analysis of Studies Conducted in the Past 10 Years |  | √ | Excluded based on title and abstract |  |
| 305 | SystEmatic review and meta-aNAlysis of infanT and young child feeding Practices (ENAT-P) in Ethiopia: protocol |  | √ | Excluded based on title and abstract |  |
| 306 | Breast and complementary feeding in Ethiopia: new national evidence from systematic review and meta-analyses of studies in the past 10 years |  | √ | Excluded based on title and abstract |  |
| 307 | Higher educational and economic status are key factors for the timely initiation of breastfeeding in Ethiopia: A review and meta-analysis |  | √ | Excluded based on title and abstract |  |
| 308 | Evidence on the effect of gender of newborn, antenatal care and postnatal care on breastfeeding practices in Ethiopia: a meta-analysis andmeta-regression analysis of observational studies |  | √ | Excluded based on title and abstract |  |
| 309 | Performance of rapid rk39 tests for the diagnosis of visceral leishmaniasis in Ethiopia: a systematic review and meta-analysis |  | √ | Excluded based on title and abstract |  |
| 310 | Burnout among Nurses Working in Ethiopia |  | √ | Excluded based on title and abstract |  |
| 311 | Prevalence of hypertension among type 2 diabetes mellitus patients in Ethiopia: a systematic review and meta-analysis |  | √ | Excluded based on title and abstract |  |
| 312 | Economic burden of adverse perinatal outcomes from births to age 5 years in high-income settings: a protocol for a systematic review |  | √ | Excluded based on title and abstract |  |
| 313 | Prevalence and Determinants of Stunting and Thinness/Wasting Among Schoolchildren of Ethiopia: A Systematic Review and Meta-Analysis |  | √ | Excluded based on title and abstract |  |
| 314 | Prevalence of Schistosoma mansoni and Associated Risk Factors in Human and Biomphalaria Snails in Ethiopia: A Systematic Review and Meta-analysis |  | √ | Excluded based on title and abstract |  |
| 315 | Risky sexual practice and associated factors among people living with HIV/AIDS receiving antiretroviral therapy in Ethiopia: Systematic review and meta-analysis |  | √ | Excluded based on title and abstract |  |
| 316 | Investigation of neuropathic pain in treated leprosy patients in Ethiopia: a cross-sectional study |  | √ | Excluded based on title and abstract |  |
| 317 | Anxiety and stress among healthcare professionals during COVID-19 in Ethiopia: systematic review and meta-analysis |  | √ | Excluded based on title and abstract |  |
| 318 | Depression and insomnia among healthcare professionals during COVID-19 pandemic in Ethiopia: a systematic review and meta-analysis |  | √ | Excluded based on title and abstract |  |
| 319 | Poor sleep quality and suicidal ideation among pregnant women during COVID-19 in Ethiopia: systematic review and meta-analysis |  | √ | Excluded based on title and abstract |  |
| 320 | Level and intensity of objectively assessed physical activity among pregnant women from urban Ethiopia |  | √ | Excluded based on title and abstract |  |
| 321 | Prevalence of Shigella species and its drug resistance pattern in Ethiopia: a systematic review and meta-analysis |  | √ | Excluded based on title and abstract |  |
| 322 | Social determinants of antenatal depression and anxiety among women in South Asia: A systematic review & meta-analysis |  | √ | Excluded based on title and abstract |  |
| 323 | Negative effects of short birth interval on child mortality in low- and middle-income countries: A systematic review and meta-analysis |  | √ | Excluded based on title and abstract |  |
| 324 | Global knowledge, attitude, and practice towards COVID-19 among pregnant women: a systematic review and meta-analysis |  | √ | Excluded based on title and abstract |  |
| 325 | Prevalence of Self-Reported Work-Related Lower Back Pain and Its Associated Factors in Ethiopia: A Systematic Review and Meta-Analysis |  | √ | Excluded based on title and abstract |  |
| 326 | Magnitude and trend of perinatal mortality and its relationship with inter-pregnancy interval in Ethiopia: a systematic review and meta-analysis |  | √ | Excluded based on title and abstract |  |
| 327 | Factors associated with modern contraceptives uptake during the first year after birth in Ethiopia: A systematic review and meta-analysis |  | √ | Excluded based on title and abstract |  |
| 328 | Identification of risk areas and practices for Taenia saginata taeniosis/cysticercosis in Ethiopia: a systematic review and meta-analysis |  | √ | Excluded based on title and abstract |  |
| 329 | Overweight and Obesity Among Adult HIV Infected Peoples Receiving ART in Ethiopia: A Systematic Review and Meta-Analysis |  | √ | Excluded based on title and abstract |  |
| 330 | Systematic review and meta-analysis of iodine deficiency and its associated factors among pregnant women in Ethiopia |  | √ | Excluded based on title and abstract |  |
| 331 | Limits and opportunities to community health worker empowerment: A multi-country comparative study |  | √ | Excluded based on title and abstract |  |
| 332 | Precancerous cervical lesion in Ethiopia: systematic review and meta-analysis |  | √ | Excluded based on title and abstract |  |
| 333 | Prevalence and factors associated with depression among older adults in the case of a low-income country, Ethiopia: a systematic review and meta-analysis |  | √ | Excluded based on title and abstract |  |
| 334 | Knowledge, attitude and practice towards antenatal physical exercise among pregnant women in Ethiopia: A systematic review and meta-analysis |  | √ | Excluded based on title and abstract |  |
| 335 | Health professionals' knowledge on vaccine cold chain management and associated factors in Ethiopia: Systematic review and meta-analysis |  | √ | Excluded based on title and abstract |  |
| 336 | Preeclampsia and its determinants in Ethiopia: A systematic review and meta-analysis |  | √ | Excluded based on title and abstract |  |
| 337 | Postpartum intrauterine contraceptive device use and its associated factors in Ethiopia: systematic review and meta-analysis |  | √ | Excluded based on title and abstract |  |
| 338 | Maternal satisfaction with emergency obstetric and newborn care services in Ethiopia and the associated factors: A systematic review and meta-analysis |  | √ | Excluded based on title and abstract |  |
| 339 | Prevalence and determinants of the involvement of married men in family planning services in Ethiopia: A systematic review and meta-analysis |  | √ | Excluded based on title and abstract |  |
| 340 | Mother-to-child transmission of HIV infection and its associated factors in Ethiopia: a systematic review and meta-analysis |  | √ | Excluded based on title and abstract |  |
| 341 | Prevalence of Violence Against Women in Ethiopia: A Meta-Analysis |  | √ | Excluded based on title and abstract |  |
| 342 | Survival of women with cervical cancer in East Africa: a systematic review and meta-analysis |  | √ | Excluded based on title and abstract |  |
| 343 | Disrespectful and abusive behavior during childbirth and maternity care in Ethiopia: a systematic review and meta-analysis |  | √ | Excluded based on title and abstract |  |
| 344 | Pooled prevalence and its determinants of stunting among children during their critical period in Ethiopia: A systematic review and meta-analysis |  | √ | Excluded based on title and abstract |  |
| 345 | Association between birth interval and wasting in children under 5 years of age in Ethiopia: a systematic review and meta-analysis protocol |  | √ | Excluded based on title and abstract |  |
| 346 | Low Economic Class Might Predispose Children under Five Years of Age to Stunting in Ethiopia: Updates of Systematic Review and Meta-Analysis |  | √ | Excluded based on title and abstract |  |
| 347 | Prevalence of overweight/obesity among the adult population in Ethiopia: a systematic review and meta-analysis |  | √ | Excluded based on title and abstract |  |
| 348 | Impact of knowledge and attitude on the utilization rate of cervical cancer screening tests among Ethiopian women: A systematic review and meta-analysis |  | √ | Excluded based on title and abstract |  |
| 349 | Breast Self-Examination Practice Among Female University Students in Ethiopia: A Systematic Review and Meta-Analysis |  | √ | Excluded based on title and abstract |  |
| 350 | Insecticide-treated bed net utilization and associated factors among pregnant women in Ethiopia: a systematic review and meta-analysis |  | √ | Excluded based on title and abstract |  |
| 351 | Minimum acceptable dietary intake among children aged 6-23 months in Ethiopia: A systematic review and meta-analysis |  | √ | Excluded based on title and abstract |  |
| 352 | Systematic review and meta-analysis of knowledge on PMTCT of HIV/AIDS and Association factors among reproductive age women in Ethiopia, 2022 |  | √ | Excluded based on title and abstract |  |
| 353 | Determinants of malnutrition among children: A systematic review |  | √ | Excluded based on title and abstract |  |
| 354 | Hepatitis B virus infection among pregnant women in Ethiopia: a systematic review and Meta-analysis of prevalence studies |  | √ | Excluded based on title and abstract |  |
| 355 | The epidemiology of syphilis in Ethiopia: a protocol for systematic review and meta-analysis covering the last three decades |  | √ | Excluded based on title and abstract |  |
| 356 | Prevalence and associated factors of early initiation of sexual intercourse among youth in Ethiopia: systematic review and meta-analysis |  | √ | Excluded based on title and abstract |  |
| 357 | Induced abortion among female students in higher education institutions in Ethiopia: A systematic review and meta-analysis |  | √ | Excluded based on title and abstract |  |
| 358 | Predictors of sexual violence among female students in higher education institutions in Ethiopia: A systematic review and meta-analysis |  | √ | Excluded based on title and abstract |  |
| 359 | Full vaccination coverage among children aged 12-23 months in Ethiopia: a systematic review and meta-analysis |  | √ | Excluded based on title and abstract |  |
| 360 | Level of self-care practice among diabetic patients in Ethiopia: a systematic review and meta-analysis |  | √ | Excluded based on title and abstract |  |
| 361 | Effects of maternal education on birth preparedness and complication readiness among Ethiopian pregnant women: a systematic review and meta-analysis |  | √ | Excluded based on title and abstract |  |
| 362 | In vivo efficacy of anti-malarial drugs against clinical Plasmodium vivax malaria in Ethiopia: a systematic review and meta-analysis |  | √ | Excluded based on title and abstract |  |
| 363 | Plasmodium vivax epidemiology in Ethiopia 2000-2020: A systematic review and meta-analysis |  | √ | Excluded based on title and abstract |  |
| 364 | Evidence on the links between water insecurity, inadequate sanitation and mental health: A systematic review and meta-analysis |  | √ | Excluded based on title and abstract |  |
| 365 | Bacterial Pathogens and Their Antimicrobial Resistance Patterns of Inanimate Surfaces and Equipment in Ethiopia: A Systematic Review and Meta-analysis |  | √ | Excluded based on title and abstract |  |
| 366 | The effect of maternal education on infant mortality in Ethiopia: A systematic review and meta-analysis |  | √ | Excluded based on title and abstract |  |
| 367 | Prevalence of polypharmacy among older adults in Ethiopia: a systematic review and meta-analysis |  | √ | Excluded based on title and abstract |  |
| 368 | Evidence-based interventions to reduce mortality among preterm and low-birthweight neonates in low-income and middle-income countries: a systematic review and meta-analysis |  | √ | Excluded based on title and abstract |  |
| 369 | Knowledge and practice of mothers towards sunshine exposure of their children in Ethiopia: a systematic review and meta-analysis |  | √ | Excluded based on title and abstract |  |
| 370 | Knowledge, attitude and practice towards COVID-19 among health professionals in Ethiopia: A systematic review and meta-analysis |  | √ | Excluded based on title and abstract |  |
| 371 | Seroprevalence of hepatitis c virus infection among blood donors in Ethiopia: a systematic review and meta-analysis |  | √ | Excluded based on title and abstract |  |
| 372 | Hypoglycemia prevention practice and associated factors among diabetes mellitus patients in Ethiopia: Systematic review and meta-analyssis |  | √ | Excluded based on title and abstract |  |
| 373 | Health-related quality of life and associated factors among cancer patients in Ethiopia: Systematic review and meta-analysis |  | √ | Excluded based on title and abstract |  |
| 374 | HIV Serostatus Disclosure and Its Predictors Among Children Living With HIV in Ethiopia: A Systematic Review and Meta-Analysis |  | √ | Excluded based on title and abstract |  |
| 375 | The impact of peer pressure on cigarette smoking among high school and university students in Ethiopia: A systemic review and meta-analysis |  | √ | Excluded based on title and abstract |  |
| 376 | Household latrine utilization and its association with educational status of household heads in Ethiopia: a systematic review and meta-analysis |  | √ | Excluded based on title and abstract |  |
| 377 | Prevalence of bovine trypanosomosis in Ethiopia: a meta-analysis |  | √ | Excluded based on title and abstract |  |
| 378 | Prevalence and Associated Risk Factors of Human Intestinal Helminths Parasitic Infections in Ethiopia: A Systematic Review and Meta-AnalysisEstimating the Intracluster Correlation Coefficient for the Clinical Sign "Trachomatous Inflammation-Follicular" in Population-Based Trachoma Prevalence Surveys: Results From a Meta-Regression Analysis of 261 Standardized Preintervention Surveys Carried Out in Ethiopia, Mozambique, and Nigeria |  | √ | Excluded based on title and abstract |  |
| 379 | Prevalence of poor sleep quality in the Ethiopian population: a systematic review and meta-analysis |  | √ | Excluded based on title and abstract |  |
| 380 | Pooled prevalence of blindness in Ethiopia: a systematic review and meta-analysis |  | √ | Excluded based on title and abstract |  |
| 381 | First line antiretroviral treatment failure and its association with drug substitution and sex among children in Ethiopia: systematic review and meta-analysis |  | √ | Excluded based on title and abstract |  |
| 382 | Parent-adolescent communication on sexual and reproductive health issues and its associated factors in Ethiopia: a systematic review and meta-analysis |  | √ | Excluded based on title and abstract |  |
| 383 | The Level of Unmet Need for Family Planning and Its Predictors among HIV-Positive Women in Ethiopia: A Systematic Review and Meta-Analysis |  | √ | Excluded based on title and abstract |  |
| 384 | Prevalence of pulmonary tuberculosis among students in three eastern Ethiopian universities |  | √ | Excluded based on title and abstract |  |
| 385 | Adolescents' Contraceptive Uptake in Ethiopia: A Meta-Analysis |  | √ | Excluded based on title and abstract |  |
| 386 | Breast self-examination practice and associated factors among female healthcare workers in Ethiopia: A systematic review and meta-analysis |  | √ | Excluded based on title and abstract |  |
| 387 | Predictors of successful vaginal birth after a cesarean section in Ethiopia: a systematic review and meta-analysis |  | √ | Excluded based on title and abstract |  |
| 388 | Routine health information utilization and associated factors among health care workers in Ethiopia: A systematic review and meta-analysis |  | √ | Excluded based on title and abstract |  |
| 389 | Prevalence and associated factors of sexual violence experienced by housemaids in Ethiopia: a systematic review and meta-analysis |  | √ | Excluded based on title and abstract |  |
| 390 | Impact of COVID-19 pandemic on utilization of essential maternal healthcare services in Ethiopia: A systematic review and meta-analysis |  | √ | Excluded based on title and abstract |  |
| 391 | Epidemiology of tuberculous lymphadenitis in Africa: A systematic review and meta-analysis |  | √ | Excluded based on title and abstract |  |
| 392 | Molecular epidemiology of M. tuberculosis in Ethiopia: A systematic review and meta-analysis |  | √ | Excluded based on title and abstract |  |
| 393 | Sero-positive HIV result disclosure to sexual partner in Ethiopia: a systematic review and meta-analysis |  | √ | Excluded based on title and abstract |  |
| 394 | Prevalence and determinants of alcohol use among adults living with HIV/AIDS in Ethiopia: a systematic review protocol |  | √ | Excluded based on title and abstract |  |
| 395 | Alcohol use and its determinants among adults living with HIV/AIDS in Ethiopia: a systematic review and meta-analysis |  | √ | Excluded based on title and abstract |  |
| 396 | Prevalence of Vancomycin resistant enterococci (VRE) in Ethiopia: a systematic review and meta-analysis |  | √ | Excluded based on title and abstract |  |
| 397 | Helicobacter pylori infections in Ethiopia; prevalence and associated factors: a systematic review and meta-analysis |  | √ | Excluded based on title and abstract |  |
| 398 | Magnitude and determinants of male partner involvement in PMTCT service utilization of pregnant women attending public health facilities of Ethiopia, 2021: a systematic review and meta-analysis |  | √ | Excluded based on title and abstract |  |
| 399 | Social phobia of Ethiopian students: meta-analysis and systematic review |  | √ | Excluded based on title and abstract |  |
| 400 | Sero-epidemiology and associated factors of HIV, HBV, HCV and syphilis among blood donors in Ethiopia: a systematic review and meta-analysis |  | √ | Excluded based on title and abstract |  |
| 401 | Male and undernourished children were at high risk of anemia in Ethiopia: a systematic review and meta-analysis |  | √ | Excluded based on title and abstract |  |
| 402 | Effect of Maternal Education on Prenatal Adherence of Iron-folic Acid Supplementation in Ethiopia: A Systematic Review and Meta-analysis |  | √ | Excluded based on title and abstract |  |
| 403 | Occupational-Related Upper and Lower Extremity Musculoskeletal Pain Among Working Population of Ethiopia: Systematic Review and Meta-Analysis |  | √ | Excluded based on title and abstract |  |
| 404 | Epidemiology of streptomycin resistant Salmonella from humans and animals in Ethiopia: A systematic review and meta-analysis |  | √ | Excluded based on title and abstract |  |
| 405 | Health related quality of life and its association with social support among people living with HIV/AIDS receiving antiretroviral therapy in Ethiopia: a systematic review and meta-analysis |  | √ | Excluded based on title and abstract |  |
| 406 | The Impact of Severe Maternal Morbidity on Perinatal Outcomes in High Income Countries: Systematic Review and Meta-Analysis |  | √ | Excluded based on title and abstract |  |
| 407 | Occupational injury and associated factors among construction workers in Ethiopia: a systematic and meta-analysis |  | √ | Excluded based on title and abstract |  |
| 408 | Diabetes mellitus among adults on highly active anti-retroviral therapy and its associated factors in Ethiopia: Systematic review and meta-analysis |  | √ | Excluded based on title and abstract |  |
| 409 | Practice and Intention to use long acting and permanent contraceptive methods among married women in Ethiopia: Systematic meta-analysis |  | √ | Excluded based on title and abstract |  |
| 410 | Magnitude of undernutrition and its association with dietary diversity among older persons in Ethiopia: a systematic review and meta-analysis, 2023 |  | √ | Excluded based on title and abstract |  |
| 411 | Active management of the third stage of labour in Ethiopia: A systematic review and meta-analysis |  | √ | Excluded based on title and abstract |  |
| 412 | Mental illness in children and its determinants in Ethiopia: A systematic review and meta-analysis, 2023 |  | √ | Excluded based on title and abstract |  |
| 413 | Prevalence of hepatotoxicity among HIV-infected patients in Ethiopia: a systematic review and meta-analysis |  | √ | Excluded based on title and abstract |  |
| 414 | Dyslipidemia among HIV-infected patients in Ethiopia: a systematic review and meta-analysis |  | √ | Excluded based on title and abstract |  |
| 415 | The state of child nutrition in Ethiopia: an umbrella review of systematic review and meta-analysis reports |  | √ | Excluded based on title and abstract |  |
| 416 | Prevalence estimates of human immunodeficiency virus (HIV) infection among visceral leishmaniasis infected people in Northwest Ethiopia: a systematic review and meta-analysis |  | √ | Excluded based on title and abstract |  |
| 417 | Cryptosporidium infection among people living with HIV/AIDS in Ethiopia: a systematic review and meta-analysis |  | √ | Excluded based on title and abstract |  |
| 418 | Prevalence of dysmenorrhea and associated factors among students in Ethiopia: A systematic review and meta-analysis |  | √ | Excluded based on title and abstract |  |
| 419 | COVID-19 vaccine acceptance among health care professionals in Ethiopia: A systematic review and meta-analysis |  | √ | Excluded based on title and abstract |  |
| 420 | Systematic review of breast and complementary feeding in Ethiopia: a commentary |  | √ | Excluded based on title and abstract |  |
| 421 | Prevalence of completion of maternity continuum of care and its associated factors in Ethiopia: a systematic review and meta-analysis |  | √ | Excluded based on title and abstract |  |
| 422 | Determinants of COVID-19 vaccine acceptance in Ethiopia: A systematic review and meta-analysis |  | √ | Excluded based on title and abstract |  |
| 423 | Pregnant women’s knowledge, attitude, and practice towards COVID-19 infection prevention in Ethiopia: A systematic review and meta-analysis |  | √ | Excluded based on title and abstract |  |
| 424 | Prevalence and determinants of risky sexual practice in Ethiopia: Systematic review and Meta-analysis |  | √ | Excluded based on title and abstract |  |
| 425 | Diabetes mellitus and its association with central obesity, and overweight/obesity among adults in Ethiopia. A systematic review and meta-analysis |  | √ | Excluded based on title and abstract |  |
| 426 | Epidemiology of preterm birth in Ethiopia: systematic review and meta-analysis |  | √ | Excluded based on title and abstract |  |
| 427 | Prevalence and associated factors of preterm birth in Ethiopia: systematic review and meta-analysis protocol |  | √ | Excluded based on title and abstract |  |
| 428 | The effect of pregnancy induced hypertension and multiple pregnancies on preterm birth in Ethiopia: a systematic review and meta-analysis |  | √ | Excluded based on title and abstract |  |
| 429 | Seroprevalence and trend of human immunodeficiency virus among blood donors in Ethiopia: a systematic review and meta-analysis |  | √ | Excluded based on title and abstract |  |
| 430 | Asthma control and its predictors in Ethiopia: Systematic review and meta-analysis |  | √ | Excluded based on title and abstract |  |
| 431 | Prevalence, antifungal susceptibility and etiology of vulvovaginal candidiasis in sub-Saharan Africa: a systematic review with meta-analysis and meta-regression |  | √ | Excluded based on title and abstract |  |
| 432 | A meta-analysis of depressive symptoms among Ethiopian prisoners and a narrative description of its associated factors: a country based systematic review and meta-analysis study |  | √ | Excluded based on title and abstract |  |
| 433 | Prevalence and determinants of maternal near miss in Ethiopia: a systematic review and meta-analysis, 2015-2023 |  | √ | Excluded based on title and abstract |  |
| 434 | Institutional delivery service utilization and associated factors in Ethiopia: a systematic review and META-analysis |  | √ | Excluded based on title and abstract |  |
| 435 | Systematic review of Ethiopian medicinal plants used for their anti-inflammatory and wound healing activities |  | √ | Excluded based on title and abstract |  |
| 436 | Tetanus Toxoid Vaccination Coverage and Associated Factors among Childbearing Women in Ethiopia: A Systematic Review and Meta-Analysis |  | √ | Excluded based on title and abstract |  |
| 437 | Magnitude of postpartum hemorrhage and its associated factors in Ethiopia: a systematic review and meta-analysis |  | √ | Excluded based on title and abstract |  |
| 438 | The impact of pastoralist mobility on tuberculosis control in Ethiopia: a systematic review and meta-synthesis |  | √ | Excluded based on title and abstract |  |
| 439 | Immunization coverage in Ethiopia among 12-23 month old children: systematic review and meta-analysis |  | √ | Excluded based on title and abstract |  |
| 440 | Predictors of immunization coverage among 12-23 month old children in Ethiopia: systematic review and meta-analysis |  | √ | Excluded based on title and abstract |  |
| 441 | Epidemiology of tungiasis in sub-saharan Africa: a systematic review and meta-analysis |  | √ | Excluded based on title and abstract |  |
| 442 | Treatment seeking delay and associated factors among tuberculosis patients attending health facility in Ethiopia from 2000 to 2020: A systematic review and meta analysis |  | √ | Excluded based on title and abstract |  |
| 443 | African Animal Trypanosomiasis: A Systematic Review on Prevalence, Risk Factors and Drug Resistance in Sub-Saharan Africa |  | √ | Excluded based on title and abstract |  |
| 444 | Birth prevalence of neural tube defects and associated risk factors in Africa: a systematic review and meta-analysis |  | √ | Excluded based on title and abstract |  |
| 445 | Biogeographical characteristics of Schistosoma mansoni endemic areas in Ethiopia: a systematic review and meta analysis |  | √ | Excluded based on title and abstract |  |
| 446 | Receptor-Defined Breast Cancer in Five East African Countries and Its Implications for Treatment: Systematic Review and Meta-Analysis |  | √ | Excluded based on title and abstract |  |
| 447 | Nasal colonization of methicillin resistant Staphylococcus aureus in Ethiopia: a systematic review and meta-analysis |  | √ | Excluded based on title and abstract |  |
| 448 | Prevalence of drug resistance-conferring mutations associated with isoniazid- and rifampicin-resistant Mycobacterium tuberculosis in Ethiopia: a systematic review and meta-analysis |  | √ | Excluded based on title and abstract |  |
| 449 | Prevalence of lifetime substances use among students in Ethiopia: a systematic review and meta-analysis |  | √ | Excluded based on title and abstract |  |
| 450 | The effects of natural disasters on leishmaniases frequency: A global systematic review and meta-analysis |  | √ | Excluded based on title and abstract |  |
| 451 | Global, regional, and national burden of cancers attributable to tobacco smoking in 204 countries and territories, 1990-2019 |  | √ | Excluded based on title and abstract |  |
| 452 | Tooth brushing practice in Ethiopia: a systematic review and meta-analysis |  | √ | Excluded based on title and abstract |  |
| 453 | Prevalence of Vitamin A Deficiency among Preschool Children in Ethiopia: A Systematic Review and Meta-Analysis |  | √ | Excluded based on title and abstract |  |
| 454 | Earning pocket money and girls' menstrual hygiene management in Ethiopia: a systematic review and meta-analysis |  | √ | Excluded based on title and abstract |  |
| 455 | Menstrual hygiene practice among adolescent girls in Ethiopia: A systematic review and meta-analysis |  | √ | Excluded based on title and abstract |  |
| 456 | The critical role of infection prevention overlooked in Ethiopia, only one-half of health-care workers had safe practice: A systematic review and meta-analysis |  | √ | Excluded based on title and abstract |  |
| 457 | Occupational exposures to blood and body fluids among healthcare workers in Ethiopia: a systematic review and meta-analysis |  | √ | Excluded based on title and abstract |  |
| 458 | Inadequate dietary diversity during pregnancy increases the risk of maternal anemia and low birth weight in Africa: A systematic review and meta‐analysis |  | √ | Excluded based on title and abstract |  |
| 459 | Adverse birth outcome and associated factors among diabetic pregnant women in Ethiopia: Systematic review and meta-analysis |  | √ | Excluded based on title and abstract |  |
| 460 | Drug-susceptible tuberculosis treatment success and associated factors in Ethiopia from 2005 to 2017: a systematic review and meta-analysis |  | √ | Excluded based on title and abstract |  |
| 461 | Adherence to iron-folic acid supplementation among pregnant women in Ethiopia: a systematic review and meta-analysis |  | √ | Excluded based on title and abstract |  |
| 462 | Preterm birth and its associated factors in Ethiopia: a systematic review and meta-analysis |  | √ | Excluded based on title and abstract |  |
| 463 | Prevalence of pressure ulcers among hospitalized adult patients in Ethiopia: a systematic review and meta-analysis |  | √ | Excluded based on title and abstract |  |
| 464 | Surgical site infection and its associated factors in Ethiopia: a systematic review and meta-analysis |  | √ | Excluded based on title and abstract |  |
| 465 | Burden and risk factors of cutaneous leishmaniasis in Ethiopia: a systematic review and meta-analysis |  | √ | Excluded based on title and abstract |  |
| 466 | A systematic review and meta-analysis on prevalence and distribution of Taenia and Echinococcus infections in Ethiopia |  | √ | Excluded based on title and abstract |  |
| 467 | Bovine tuberculosis in Ethiopia: A systematic review and meta-analysis |  | √ | Excluded based on title and abstract |  |
| 468 | Brucellosis in Ethiopia: A comprehensive review of literature from the year 2000-2020 and the way forward |  | √ | Excluded based on title and abstract |  |
| 469 | Environmental and life-style risk factors for esophageal squamous cell carcinoma in Africa: a systematic review and meta-analysis |  | √ | Excluded based on title and abstract |  |
| 470 | Epidemiology of self-medication in Ethiopia: a systematic review and meta-analysis of observational studies |  | √ | Excluded based on title and abstract |  |
| 471 | Resistance profile of clinically relevant bacterial isolates against fluoroquinolone in Ethiopia: a systematic review and meta-analysis |  | √ | Excluded based on title and abstract |  |
| 472 | Microbial epidemiology and antimicrobial resistance patterns of wound infection in Ethiopia: a meta-analysis of laboratory-based cross-sectional studies |  | √ | Excluded based on title and abstract |  |
| 473 | Food handling practice and associated factors among food handlers in public food establishments of Ethiopia: a systematic review and meta-analysis |  | √ | Excluded based on title and abstract |  |
| 474 | Routine health information use among healthcare providers in Ethiopia: a systematic review and meta-analysis |  | √ | Excluded based on title and abstract |  |
| 475 | Vitamin D deficiency and its associated factors among patients with type 2 diabetes mellitus: a systematic review and meta-analysis |  | √ | Excluded based on title and abstract |  |
| 476 | Anemia and its predictors among chronic kidney disease patients in Sub-Saharan African countries: A systematic review and meta-analysis |  | √ | Excluded based on title and abstract |  |
| 477 | Determinants of birth asphyxia among preterm newborns in Ethiopia: a systematic review and meta-analysis of observational studies protocol |  | √ | Excluded based on title and abstract |  |
| 478 | Malaria and Helminthic Co-Infection during Pregnancy in Sub-Saharan Africa: A Systematic Review and Meta-Analysis |  | √ | Excluded based on title and abstract |  |
| 479 | Diabetic Peripheral Neuropathy in Ethiopia: A Systematic Review and Meta-Analysis |  | √ | Excluded based on title and abstract |  |
| 480 | Self-care practice among hypertensive patients in Ethiopia: systematic review and meta-analysis |  | √ | Excluded based on title and abstract |  |
| 481 | Uncontrolled asthma in Ethiopia: a systematic review and meta-analysis |  | √ | Excluded based on title and abstract |  |
| 482 | Prevalence of human Salmonellosis in Ethiopia: a systematic review and meta-analysis |  | √ | Excluded based on title and abstract |  |
| 483 | A meta-analysis of the proportion of antimicrobial resistant human Salmonella isolates in Ethiopia |  | √ | Excluded based on title and abstract |  |
| 484 | A meta-analysis of the proportion of animal Salmonella isolates resistant to drugs used against human salmonellosis in Ethiopia |  | √ | Excluded based on title and abstract |  |
| 485 | Brucellosis Seropositivity in Animals and Humans in Ethiopia: A Meta-analysis |  | √ | Excluded based on title and abstract |  |
| 486 | Prevalence of Salmonella in raw animal products in Ethiopia: a meta-analysis |  | √ | Excluded based on title and abstract |  |
| 487 | A meta-analysis of the prevalence of Salmonella in food animals in Ethiopia |  | √ | Excluded based on title and abstract |  |
| 488 | Framework for maternal morbidity and mortality interventions in Ethiopia: a systematic review protocol |  | √ | Excluded based on title and abstract |  |
| 489 | Undernutrition and its determinants among Ethiopian adolescent girls: a protocol for systematic review and meta-analysis |  | √ | Excluded based on title and abstract |  |
| 490 | Food safety practice and associated factors in public food establishments of Ethiopia: A systematic review and meta-analysis |  | √ | Excluded based on title and abstract |  |
| 491 | Water treatment at the point-of-use and treatment preferences among households in Ethiopia: A contemporaneous systematic review and meta-analysis |  | √ | Excluded based on title and abstract |  |
| 492 | Attack rate, case fatality rate and determinants of measles infection during a measles outbreak in Ethiopia: systematic review and meta-analysis |  | √ | Excluded based on title and abstract |  |
| 493 | The risk of mother-to-child transmission of hepatitis B virus infection in Ethiopia: A systematic review and meta-analysis |  | √ | Excluded based on title and abstract |  |
| 494 | The effect of gestational age, low birth weight and parity on birth asphyxia among neonates in sub-Saharan Africa: systematic review and meta-analysis: 2021 |  | √ | Excluded based on title and abstract |  |
| 495 | Tuberculosis Treatment Outcome and Predictors in Africa: A Systematic Review and Meta-Analysis |  | √ | Excluded based on title and abstract |  |
| 496 | Prevalence and factors associated with hypertension among peoples living with HIV in East Africa, a systematic review and meta-analysis |  | √ | Excluded based on title and abstract |  |
| 497 | Depression and determinants among diabetes mellitus patients in Ethiopia, a systematic review and meta-analysis |  | √ | Excluded based on title and abstract |  |
| 498 | Prevalence of urinary tract infections and risk factors among diabetic patients in Ethiopia, a systematic review and meta-analysis |  | √ | Excluded based on title and abstract |  |
| 499 | Prevalence of central obesity and associated factors in Ethiopia: A systematic review and meta-analysis |  | √ | Excluded based on title and abstract |  |
| 500 | Neonatal mortality in Ethiopia: a protocol for systematic review and meta-analysis |  | √ | Excluded based on title and abstract |  |
| 501 | Factors affecting utilization of antenatal care in Ethiopia: A systematic review and meta-analysis |  | √ | Excluded based on title and abstract |  |
| 502 | Dietary diversity feeding practice and its associated factors among children age 6-23 months in Ethiopia from 2011 up to 2018: a systematic review and meta-analysis |  | √ | Excluded based on title and abstract |  |
| 503 | Assessment of Place of Delivery and Associated Factors among Pastoralists in Ethiopia: A Systematic Review and Meta-Analysis Evaluation |  | √ | Excluded based on title and abstract |  |
| 504 | Assessment of medicine use among outpatients at healthcare facilities in Ethiopia using the WHO's prescribing indicators with a focus on antibiotics: a systematic review and meta-analysis |  | √ | Excluded based on title and abstract |  |
| 505 | Systematic review and meta-analysis of medicine use studies in Ethiopia using the WHO patient care indicators with an emphasis on the availability of prescribed medicines |  | √ | Excluded based on title and abstract |  |
| 506 | Prevalence and determinants of hypertensive disorders of pregnancy in Ethiopia: A systematic review and meta-analysis |  | √ | Excluded based on title and abstract |  |
| 507 | The Magnitude of NCD Risk Factors in Ethiopia: Meta-Analysis and Systematic Review of Evidence |  | √ | Excluded based on title and abstract |  |
| 508 | Prevalence of chronic non-communicable diseases in Ethiopia: A systematic review and meta-analysis of evidence |  | √ | Excluded based on title and abstract |  |
| 509 | Birth prevalence and risk factors of neural tube defects in Ethiopia: a systematic review and meta-analysis |  | √ | Excluded based on title and abstract |  |
| 510 | The twin epidemics: Prevalence of TB/HIV co-infection and its associated factors in Ethiopia; A systematic review and meta-analysis |  | √ | Excluded based on title and abstract |  |
| 512 | Diabetes Mellitus and Its Association with Hypertension in Ethiopia: A Systematic Review and Meta-Analysis |  | √ | Excluded based on title and abstract |  |
| 513 | Chronic Liver Disease in Ethiopia with a Particular Focus on the Etiological Spectrums: A Systematic Review and Meta-Analysis of Observational Studies |  | √ | Excluded based on title and abstract |  |
| 514 | Delayed initiation of antenatal care and associated factors in Ethiopia: a systematic review and meta-analysis |  | √ | Excluded based on title and abstract |  |
| 515 | The Prevotella copri Complex Comprises Four Distinct Clades Underrepresented in Westernized Populations |  | √ | Excluded based on title and abstract |  |
| 516 | Tuberculosis-Human Immunodeficiency Virus (HIV) co-infection in Ethiopia: a systematic review and meta-analysis |  | √ | Excluded based on title and abstract |  |
| 517 | Prevalence of anemia among school-age children in Ethiopia: a systematic review and meta-analysis |  | √ | Excluded based on title and abstract |  |
| 518 | Tuberculosis infection control practice among healthcare workers in Ethiopia: a protocol for systematic review and meta-analysis |  | √ | Excluded based on title and abstract |  |
| 519 | Tuberculosis infection control practice and associated factors among health care workers in Ethiopia: Systematic review and meta-analysis |  | √ | Excluded based on title and abstract |  |
| 520 | Prevalence of hypertension and its determinants in Ethiopia: A systematic review and meta-analysis |  | √ | Excluded based on title and abstract |  |
| 521 | Antenatal Care Reduces Neonatal Mortality in Ethiopia: A Systematic Review and Meta-Analysis of Observational Studies |  | √ | Excluded based on title and abstract |  |
| 522 | Prevalence of tuberculosis treatment non-adherence in Ethiopia: a systematic review and meta-analysis |  | √ | Excluded based on title and abstract |  |
| 523 | Dropout rate and associated factors of community-based health insurance beneficiaries in Ethiopia: a systematic review and meta-analysis |  | √ | Excluded based on title and abstract |  |
| 524 | Magnitude of postpartum hemorrhage and associated factors among women who gave birth in Ethiopia: a systematic review and meta-analysis |  | √ | Excluded based on title and abstract |  |
| 525 | Prevalence and associated factors of foot ulcer among diabetic patients in Ethiopia: a systematic review and meta-analysis |  | √ | Excluded based on title and abstract |  |
| 526 | Association between pregnancy intention and late initiation of antenatal care among pregnant women in Ethiopia: a systematic review and meta-analysis |  | √ | Excluded based on title and abstract |  |
| 527 | Bacterial profile of bovine mastitis in Ethiopia: a systematic review and meta-analysis |  | √ | Excluded based on title and abstract |  |
| 528 | Precancerous lesions of the cervix and its determinants among Ethiopian women: Systematic review and meta-analysis |  | √ | Excluded based on title and abstract |  |
| 529 | Prevalence of herbal and traditional medicine in Ethiopia: a systematic review and meta-analysis of 20-year studies |  | √ | Excluded based on title and abstract |  |
| 530 | Drug Resistance Patterns of Escherichia coli in Ethiopia: A Meta-Analysis |  | √ | Excluded based on title and abstract |  |
| 531 | High rate of extended-spectrum beta-lactamase-producing gram-negative infections and associated mortality in Ethiopia: a systematic review and meta-analysis |  | √ | Excluded based on title and abstract |  |
| 532 | Comments on the published meta-analysis and systematic review of the prevalence of multidrug-resistant bacteria in Ethiopia |  | √ | Excluded based on title and abstract |  |
| 533 | A meta-analysis of inpatient treatment outcomes of severe acute malnutrition and predictors of mortality among under-five children in Ethiopia |  | √ | Excluded based on title and abstract |  |
| 534 | Burden of anemia and its association with HAART in HIV infected children in Ethiopia: a systematic review and meta-analysis |  | √ | Excluded based on title and abstract |  |
| 535 | An overview of the case fatality of inpatient severe acute malnutrition in Ethiopia and its association with human immunodeficiency virus/tuberculosis comorbidity-a systematic review and meta-analysis |  | √ | Excluded based on title and abstract |  |
| 536 | A Systematic Review and Meta-Analysis on Post-Abortion Contraceptive Utilization and Associated Factors in Ethiopia |  | √ | Excluded based on title and abstract |  |
| 537 | Prevalence of exclusive breastfeeding practice and its association with maternal employment in Ethiopia: a systematic review and meta-analysis |  | √ | Excluded based on title and abstract |  |
| 538 | Postpartum modern contraception utilization and its determinants in Ethiopia: A systematic review and meta-analysis |  | √ | Excluded based on title and abstract |  |
| 539 | Exploring facilitators and barriers of the sustainable acceptance of e-health system solutions in Ethiopia: A systematic review |  | √ | Excluded based on title and abstract |  |
| 540 | Prevalence of erectile dysfunction and its associated factors among patients with diabetes in Ethiopia: a systematic review and meta-analysis |  | √ | Excluded based on title and abstract |  |
| 541 | Knowledge, attitudes and prevention practices related to dog-mediated rabies in Ethiopia: a systematic review and meta-analysis of observational epidemiological studies from inception to 2023 |  | √ | Excluded based on title and abstract |  |
| 542 | A systematic review and meta-analysis of antimicrobial resistance knowledge, attitudes, and practices: Current evidence to build a strong national antimicrobial drug resistance narrative in Ethiopia |  | √ | Excluded based on title and abstract |  |
| 543 | Intestinal parasitic infections and associated factors among people living with HIV/AIDS in Ethiopia: A systematic review and meta-analysis |  | √ | Excluded based on title and abstract |  |
| 544 | Self-care behaviour and associated factors among heart failure patients in Ethiopia: a systematic review and meta-analysis |  | √ | Excluded based on title and abstract |  |
| 545 | Household satisfaction and associated factors with community-based health insurance scheme in Ethiopia: systematic review and meta-analysis |  | √ | Excluded based on title and abstract |  |
| 546 | Prevalence of sexual violence in Ethiopian workplaces: systematic review and meta-analysis |  | √ | Excluded based on title and abstract |  |
| 547 | Effects of vitamin D on neonatal sepsis: A systematic review and meta‐analysis |  | √ | Excluded based on title and abstract |  |
| 548 | eHealth literacy and its associated factors in Ethiopia: Systematic review and meta-analysis |  | √ | Excluded based on title and abstract |  |
| 549 | Menstrual hygiene practice among female adolescents and its association with knowledge in Ethiopia: A systematic review and meta-analysis |  | √ | Excluded based on title and abstract |  |
| 550 | The effect of counseling, antiretroviral therapy and relationship on disclosing HIV positive status to sexual partner among adult HIV patients in Ethiopia: A systematic review and meta-analysis |  | √ | Excluded based on title and abstract |  |
| 551 | Recovery rate and determinants of severe acute malnutrition children treatment in Ethiopia: a systematic review and meta-analysis |  | √ | Excluded based on title and abstract |  |
| 552 | Prevalence of needlestick injury among healthcare workers in Ethiopia: a systematic review and meta-analysis |  | √ | Excluded based on title and abstract |  |
| 553 | An updated systematic review and meta-analysis of the prevalence of hepatitis B virus in Ethiopia |  | √ | Excluded based on title and abstract |  |
| 554 | Treatment Outcome of Epileptic Patients Receiving Antiepileptic Drugs in Ethiopia: A Systematic Review and Meta-Analysis |  | √ | Excluded based on title and abstract |  |
| 555 | Should statin guidelines consider patient preferences? Eliciting preferences of benefit and harm outcomes of statins for primary prevention of cardiovascular disease in the sub-Saharan African and European contexts |  | √ | Excluded based on title and abstract |  |
| 556 | Human immunodeficiency virus positive status disclosure to a sexual partner and its determinant factors in Ethiopia: a systematic review and meta-analysis |  | √ | Excluded based on title and abstract |  |
| 557 | Do adult patients with diabetes mellitus living in rural part of Ethiopia and having poor social support have disproportionately poor self-care practice? A systematic review and meta-analysis |  | √ | Excluded based on title and abstract |  |
| 558 | Obstructed labor and its effect on adverse maternal and fetal outcomes in Ethiopia: A systematic review and meta-analysis |  | √ | Excluded based on title and abstract |  |
| 559 | Obstructed labor and its association with adverse feto-maternal outcome in Ethiopia: a protocol for a systematic review and meta-analysis |  | √ | Excluded based on title and abstract |  |
| 560 | Breast self-examination practice and its determinants among women in Ethiopia: A systematic review and meta-analysis |  | √ | Excluded based on title and abstract |  |
| 561 | Intestinal parasites among food handlers of food service establishments in Ethiopia: a systematic review and meta-analysis |  | √ | Excluded based on title and abstract |  |
| 562 | Undernutrition and associated factors among older adults in Ethiopia: systematic review and meta-analysis |  | √ | Excluded based on title and abstract |  |
| 563 | Undiagnosed diabetes mellitus and associated factors among adults in Ethiopia: a systematic review and meta-analysis |  | √ | Excluded based on title and abstract |  |
| 564 | Prevalence and determinants of antenatal depression among pregnant women in Ethiopia: a systematic review and meta-analysis |  | √ | Excluded based on title and abstract |  |
| 565 | Prevalence and determinants of anti-tuberculosis treatment non-adherence in Ethiopia: A systematic review and meta-analysis |  | √ | Excluded based on title and abstract |  |
| 566 | Epidemiology of Newcastle disease in chickens of Ethiopia: a systematic review and meta-analysis |  | √ | Excluded based on title and abstract |  |
| 567 | Prevalence and associated factors of post-partum depression in Ethiopia. A systematic review and meta-analysis |  | √ | Excluded based on title and abstract |  |
| 568 | Prevalence and Associated Factors of Precancerous Cervical Lesions among Women in Ethiopia: A Systematic Review and Meta-Analysis |  | √ | Excluded based on title and abstract |  |
| 569 | Quality of adolescent and youth-friendly sexual and reproductive health services and associated factors in Ethiopia: a systematic review and meta-analysis |  | √ | Excluded based on title and abstract |  |
| 570 | Level of Mothers'/Caregivers' Healthcare-Seeking Behavior for Child's Diarrhea, Fever, and Respiratory Tract Infections and Associated Factors in Ethiopia: A Systematic Review and Meta-Analysis |  | √ | Excluded based on title and abstract |  |
| 571 | Prevalence of Campylobacter species in human, animal and food of animal origin and their antimicrobial susceptibility in Ethiopia: a systematic review and meta-analysis |  | √ | Excluded based on title and abstract |  |
| 572 | A systematic review and meta-analysis of HIV associated neurocognitive disorders (HAND) among people with HIV in Ethiopia |  | √ | Excluded based on title and abstract |  |
| 573 | Prevalence and risk factors of type-2 diabetes mellitus in Ethiopia: systematic review and meta-analysis |  | √ | Excluded based on title and abstract |  |
| 574 | Determinants of evidence-based practice among health care professionals in Ethiopia: A systematic review and meta-analysis |  | √ | Excluded based on title and abstract |  |
| 575 | Human papillomavirus vaccine acceptance among adolescent girls in Ethiopia: a systematic review and meta-analysis |  | √ | Excluded based on title and abstract |  |
| 576 | Essential maternal health service disruptions in Ethiopia during COVID 19 pandemic: a systematic review |  | √ | Excluded based on title and abstract |  |
| 577 | Determinants of late-stage cervical cancer presentation in Ethiopia: a systematic review and meta-analysis |  | √ | Excluded based on title and abstract |  |
| 578 | Dental caries and associated factors in Ethiopia: systematic review and meta-analysis |  | √ | Excluded based on title and abstract |  |
| 579 | A review of low birth weight in Ethiopia: socio-demographic and obstetric risk factors |  | √ | did not meet the inclusion criteria(narrative) |  |
| 580 | Neonatal hypothermia and its association with delayed initiation of breastfeeding and low birth weight in Ethiopia: Systematic review and meta-analysis |  | √ | didn't consider the required outcome |  |
| 581 | The Burden of Adverse Neonatal Outcome among Antenatal Substance Users in Ethiopia: A Systematic Review and Meta-Analysis |  | √ | conducted among high risk populations |  |
| 582 | The effect of substance use during pregnancy on neonatal outcomes in Ethiopia: A systematic review and meta-analysis | √ |  |  |  |
| 583 | A systematic study and meta-analysis: the prevalence of low birth weight and its determinants in Ethiopia | √ |  |  | https://papers.ssrn.com/sol3/papers.cfm?abstract_id=4417075 |
| 584 | Intimate partner violence during pregnancy and adverse birth outcomes in Ethiopia: A systematic review and meta-analysis | √ |  |  |  |
| 585 | Prevalence and Determinants of Low Birth Weight in Ethiopia: A Systematic Review and Meta-Analysis | √ |  |  |  |
| 586 | Environmental exposures and adverse pregnancy outcomes in Ethiopia: A systematic review and meta-analysis | √ |  |  |  |
| 587 | Low birth weight and its associated factors in Ethiopia: a systematic review and meta-analysis | √ |  |  |  |
| 588 | Adverse fetal outcomes and its associated factors in Ethiopia: a systematic review and meta-analysis | √ |  |  |  |
| 589 | The impact of pregnancy induced hypertension on low birth weight in Ethiopia: systematic review and meta-analysis | √ |  |  |  |
| 590 | Prevalence and Determinants of Low Birth Weight in Ethiopia: A Systematic Review and Meta-Analysis | √ |  |  |  |
| 591 | Obstetric emergencies and adverse maternal-perinatal outcomes in Ethiopia; A systematic review and meta-analysis |  | √ | didn't consider the required outcome |  |
| 592 | Maternal and perinatal outcomes of hypertensive disorders of pregnancy in Ethiopia: systematic review and meta-analysis |  | √ | conducted among high risk populations |  |
| 593 | The effect of antenatal care on perinatal outcomes in Ethiopia: A systematic review and meta-analysis |  | √ | didn't consider the required outcome |  |
| 594 | Iron with folic acid supplemenation and birth weight in Ethiopia: Systemic review and meta-analysis | √ |  |  |  |
| 595 | Relationship between periodontal disease and preterm low birth weight: systematic review |  | √ | didn't explain clearly the required outcome of interest |  |
| 596 | Association between iron and folic acid supplementation and birth weight in Ethiopia: systemic review and meta analysis | √ |  |  |  |
