# Supplementary material for: Prevalence of low birth weight and associated factors in Ethiopia: An umbrella review of systematic review and meta-analyses
Source: PLOS Glob Public Health. 2025 May 8;5(5):e0004556. doi: 10.1371/journal.pgph.0004556 (PMC12061095; doi:10.1371/journal.pgph.0004556)
Supplement: S3 Table — (DOCX) [file pgph.0004556.s005.docx]

Quality assessment of included SRMA studies using AMSTAR-2

| S.no | Authors | Q1 | Q2 | Q3 | Q4 | Q5 | Q6 | Q7 | Q8 | Q9 | Q10 | Q11 | Q12 | Q13 | Q14 | Q15 | Q16 | Quality score |
| --- | --- | --- | --- | --- | --- | --- | --- | --- | --- | --- | --- | --- | --- | --- | --- | --- | --- | --- |
| 1 | Katiso NA et al. | 2 | 1 | 2 | 2 | 2 | 2 | 2 | 2 | 1 | 0 | 2 | 0 | 2 | 2 | 2 | 2 | Medium |
| 2 | Gedefaw G et al. | 2 | 2 | 2 | 2 | 2 | 2 | 2 | 2 | 2 | 2 | 2 | 2 | 2 | 2 | 2 | 2 | High |
| 3 | Endalamaw A et al. | 2 | 2 | 2 | 2 | 2 | 2 | 2 | 2 | 2 | 2 | 2 | 2 | 2 | 2 | 2 | 2 | High |
| 4 | Getaneh T et al. | 2 | 2 | 2 | 2 | 2 | 2 | 2 | 2 | 2 | 2 | 2 | 2 | 2 | 2 | 2 | 2 | High |
| 5 | Samuel DH,et al. | 2 | 1 | 2 | 2 | 2 | 2 | 1 | 2 | 1 | 0 | 2 | 0 | 2 | 2 | 2 | 2 | Medium |
| 6 | Wubet AB.et al. | 2 | 1 | 2 | 2 | 2 | 2 | 2 | 2 | 2 | 2 | 2 | 2 | 2 | 2 | 2 | 2 | High |
| 7 | Tegegne K. T. et al. | 2 | 1 | 2 | 1 | 2 | 2 | 1 | 2 | 1 | 0 | 2 | 0 | 0 | 2 | 2 | 2 | Critically low |
| 8 | Zenebe et al. | 2 | 1 | 2 | 2 | 2 | 2 | 1 | 2 | 1 | 2 | 2 | 0 | 0 | 2 | 2 | 2 | Critically low |
| 9 | TamiratD.et al. | 2 | 2 | 2 | 2 | 2 | 2 | 2 | 2 | 1 | 0 | 2 | 0 | 2 | 2 | 2 | 2 | Medium |
| 10 | Belay HG,et al. | 2 | 1 | 2 | 2 | 2 | 2 | 2 | 2 | 1 | 2 | 2 | 0 | 2 | 2 | 2 | 2 | Medium |
| 11 | Demelash EH, et al. | 2 | 2 | 2 | 2 | 2 | 2 | 2 | 2 | 2 | 2 | 2 | 2 | 2 | 2 | 2 | 2 | High |
