# Supplementary material for: Prevalence of low birth weight and associated factors in Ethiopia: An umbrella review of systematic review and meta-analyses
Source: PLOS Glob Public Health. 2025 May 8;5(5):e0004556. doi: 10.1371/journal.pgph.0004556 (PMC12061095; doi:10.1371/journal.pgph.0004556)
Supplement: Attachment — Submitted filename: Response to reviewer.docx [file pgph.0004556.s007.docx]

**POINT-BY-POINT REBUTTAL LETTER**

**Comments to the Author**

**Reviewer #1**
 Methods

1. Eligibility criteria:

- Line 99-100: It will be good if the authors offered an explanation as to why there were no restrictions to publication dates in their search strategy.

***Thank you for your concerns, dear reviewer. We believe there is limited data on this topic, which is why we do not emphasize the study period to include a greater number of studies.***

2. Data extraction:

- Did the standardized data extraction form have a section summarizing the author’s overall conclusions? And if not, why?

***Thank you again dear reviewer, yes the authors main findings were summarized in the table which is attached as supplementary file 1.***

- How many studies would have been required to have carried out an assessment of publication bias?
***Thank you for your question, to assess publication bias, at least ten studies were required.***

Results

1. Characteristics of included systematic review and meta-analysis studies:

- The authors present participants with primary outcome of interest on line 170 then present participants with outcomes of interest. It would be clearer if the authors specify which outcomes of interest they are presenting. The same goes for the number of studies with primary outcome and number of studies with outcomes of interest.

***Thank you very much for your constructive comments. Accepted comments and revision was made in the main document.***

- Line 179: Authors to consider placing the references at the end of the sentence.

***Accepted comments and correction was made in the main document.***

- Even though there were no date restrictions for the search strategy, it will be important for the authors to state the time span that their chosen SRMAs covered.

***Thank you very much accepted your comments and revision was made***

What is missing from this section is a discussion on the results of the quality assessments of the SMRAs chosen using the AMSTAR-2 tool.

***Thank you very much for your comments. However, we put the details of the quality assessments of the SRMAs using the AMSTAR-2 tool under sub-heading (Quality assessment of the systematic review and meta-analyzed studies). The details of the result were attached as supplementary file.***

2. Primary studies

- The explanations were not very clear here. For example, when discussing the SRMAs that overlapped, did you mean to say that the 6 SRMAs that included one primary study had 1 primary study in common to all of them?

***Thank you very much for your concern. Here to make it clear, a total of 226 primary studies were included in the 11 SRMA studies reported LBW in this umbrella review. Of the 11 SRMA studies included in this review six SRMA studies have had one primary study in common.***

It will also be important to include the reference to this common primary study (ies).

***Accepted comments and revision was made.***

Furthermore, when discussing the SRMAs that had no overlap, even though you have referenced them by author name, please add their respective reference numbers.

***Accepted comments and revision was made.***

- Line 196-205: The authors may want to consider breaking down this statement into shorter sentences as it’s quite long in its current state and not easily understood.

***Accepted comments and revision was made.***

3. Meta-analysis of predictors of LBW

- The authors may want to consider adding a subtitle to each of the paragraphs with the predictor of interest being discussed.

***Thank you very much dear reviewer for your suggestion. Accepted comments and revision was made.***

- Again, please add reference numbers to the papers you are citing.

***Accepted comments and revision was made.***

- Line 250-251: What was the pregnancy interval considered by Habtegiorgis et al?

***Accepted and revision was made.***

- Line 255: Please define IFAS as this is the first time the authors are using it in the main paper.

***Accepted comments and revision was made.***

- Line 264-265: What was the maternal age considered by Habtegiorgis et al?

***Accepted and revision was made.***

Discussion
- Line 286-291: There is a correlation between LBW and prematurity. It is easier to make the argument of the LBW due to prematurity because the neonate is expected to have a weight lower than 2500g when they are not at term. But no so much for the maturity of the organs.

***Thank you for your concern and revision was made.***

- Line 299: “Therefore, particular emphasis shall be given to scaling up regular antenatal care
follow up, health education, early detection, and intervention of obstetric complications with the help of Community Health Workers/ Volunteers (CHW/Vs).”. Were the authors intending to give a recommendation here (and not so much what they will be doing following the release of the paper)?

If the former, I propose they change ‘shall’ to ‘should’.

***Accepted and revision was made***

- Line 298: The paper being cited for LMICs (Khanal et al) did not look at LMICS in general but specifically studied Nepal.

***Accepted and revision was made***

- Line 317-320: The authors highlight that short pregnancy intervals can result in LBW. But would inadequate maternal intake or depletion of iron and folate alone have a direct significant impact on maternal weight?

***Thank you very much for your concerns as we put in the main document inadequate maternal intake or depletion of iron and folate as a result of short pregnancy interval are underlying risk factors that may increases the risk of anemia and compromises their nutritional status, which has a negative impact on the growth of the fetus.***

***However, we accept your comments and revision was made.***

Limitations
- This section would benefit from a more nuanced discussion on what types of heterogeneity the authors observed that could be a limitation to this study.

***Thank you dear reviewer for your concerns, we report heterogeneity as a limitation to this study that may result from included SRMA studies use different source population to study the outcome variable that may have introduced some heterogeneity, as would the use of different study design among primary studies.***

**Reviewer #2:**

Thank you for the opportunity to review this important manuscript. Understanding the prevalence of LBW in Ethiopia is important for informing efforts to address it. The authors have done a very thorough review of the literature, and the manuscript provides an in-depth discussion of factors that put newborns at higher risk of LBW.

I have several suggestions for strengthening the manuscript:

1) My main concern is about the overlap in the primary studies included in the systematic reviews. If I understand correctly, the sum of the number of primary studies across systematic reviews was 259, but only 79 of these were unique, indicating a very high level of overlap. While the authors acknowledge this issue, they still use statistical methods that treat each of the 259 studies as unique. This results in a false level of precision, and also biases the findings towards studies that appear more times in systematic reviews. To make these findings interpretable, I think it will be important to conduct an analysis that removes duplicates.

***Thank you, dear reviewer, for clarifying that out of 259 studies, only 226 reported LBW. We identified seventy-nine distinct primary publications, suggesting that at least two SRMA studies shared primary studies, which the authors recognize as a drawback of the umbrella review. Additionally, our statistical analysis using corrected covered area (CCA) methods revealed a moderate overlap.***

**Statistical approach**

**c= number of included SR (Number of columns)**

**r= number of publications of primary studies (Number of rows)**

**N= number of total primary studies included in SR, including double counting**

**Corrected covered area CCA = (N-r)/ (rc-r)**

**Interpretation**

**0-5% =slight over lap**

**6-10%= moderate over lap**

**11-15%= high overlap**

**>15% very high over lap**

| **Number of included SR (Number of columns)** | **c** | **11** |
| --- | --- | --- |
| **Number of publications of primary studies (Number of rows)** | **r** | **134** |
| **Number of total primary studies included in SR, (including double counting)** | **N** | **226** |
| **Covered area** | **N/(rc)= 226/(134*11)** | **15.3%** |
| **Corrected covered area** | **(N-r)/(rc-r)= (226-134)/(134*11-134)** | **6.86%** |
| **Interpretation of overlap** | **Moderate over lap** | |

My other comments are minor:

2) In the introduction, the authors quote a statistic that the global prevalence of LBW is 15.5%, but they also state that the prevalence is 9.8% in sub-Saharan Africa. These numbers come very different sources, so it is hard to compare them. I would recommend using one consistent source for all estimates, such as Okwaraji et al 2024 in the Lancet (National, regional, and global estimates of low birthweight in 2020, with trends from 2000: a systematic analysis) - that study estimates 16.6% global prevalence and 15.7% prevalence in sub-Saharan Africa.

***Thank you very much accepted comments and revision was made.***

3) In the discussion section, the authors talk about the reasons for the association between prematurity and low birth weight. One reason that is not mentioned is that premature babies have less time in utero and therefore less time to gain weight.

***Thank you again for your constructive suggestion. Accepted and revision was made.***

4) In the discussion section, the authors also talk about the relationship between IFA and low birth weight. I would recommend adding a point that this association may be partly driven by unmeasured confounding, e.g. by socioeconomic status.

***Thank you dear reviewer.Yes of course, women from lower socioeconomic backgrounds may have limited access to prenatal care and nutritional resources, which could contribute to both lower rates of IFA supplementation and higher incidences of low birth weight. However, in the primary study and systematic review authors may control this unmeasured confounding variable and report their result.***

5) There are some minor grammatical/language errors throughout the manuscript (e.g., the phrase "limited convulsive information" in the introduction)

***Thank you revision was made.***
