# Supplementary material for: Prevalence of low birth weight and associated factors in Ethiopia: An umbrella review of systematic review and meta-analyses
Source: PLOS Glob Public Health. 2025 May 8;5(5):e0004556. doi: 10.1371/journal.pgph.0004556 (PMC12061095; doi:10.1371/journal.pgph.0004556)
Supplement: Attachment — Submitted filename: response_to_reviewer_auresp_2.docx [file pgph.0004556.s008.docx]

**POINT-BY-POINT REBUTTAL LETTER**

**Comments to the Author**

**Reviewer # 2**

1 Missing data analysis - Can you please provide more detailed information on the sensitivity analyses conducted for missing data?

*Authors’ response: Thank you dear reviewer, as you know we do not have any missing data however, to assess how robust the results are to various assumptions or decisions, including how to handle missing data, the authors have checked robustness of the finding by alternatively changing of effect sizes, fixed and random models, removing studies with large or small sample sizes. No single study significantly affected the overall pooled estimate.*

2 Overlap - In the analysis of overlap, you currently use r=134. However, in the text of the manuscript, you say that r=79 (there are 79 unique primary publications). Which is correct? If you do the calculation for CCA using r=79, the result is 18.6, which indicates avert high degree of overlap. Please clarify this in the next version.

**Authors’ response**: *Thank you for the concern. r = number of publications of primary studies (Number of rows)) means those primary studies included in the 11 SRMA studies which means 134 studies, so r means not a unique primary publication rather it is the overall studies included in the 11 SRMA studies. Out of 134 studies included in the 11 SRMA 79 are unique while 55 studies were repeated in either of the 11 SRMA studies .Those unique studies (79 studies) plus 55 repeated studies in the either of 11 SRMA studies gives* N= number of total primary studies included in SR, including double counting *(226 studies). The authors attached the list of those studies as supplementary file.*

Statistical approach

c= number of included SR (Number of columns)

r= number of publications of primary studies (Number of rows)

N= number of total primary studies included in SR, including double counting

**Corrected covered area CCA = (N-r)/ (rc-r)**

**Interpretation**

**0-5% =slight over lap**

**6-10%= moderate over lap**

**11-15%= high overlap**

**>15% very high over lap**

| **Number of included SR (Number of columns)** | **C** | **11** |
| --- | --- | --- |
| **Number of publications of primary studies (Number of rows)** | **R** | **134** |
| **Number of total primary studies included in SR, (including double counting)** | **N** | **226** |
| **Covered area** | **N/(rc)= 226/(134*11)** | **15.3%** |
| **Corrected covered area** | **(N-r)/(rc-r)= (226-134)/(134*11-134)** | **6.86%** |
| **Interpretation of overlap** | **Moderate over lap** | |
