# Supplementary material for: Prevalence of low birth weight and associated factors in Ethiopia: An umbrella review of systematic review and meta-analyses
Source: PLOS Glob Public Health. 2025 May 8;5(5):e0004556. doi: 10.1371/journal.pgph.0004556 (PMC12061095; doi:10.1371/journal.pgph.0004556)
Supplement: Attachment — Submitted filename: Review Comments][Non 2, 2024.doc [file pgph.0004556.s009.doc]

**REVIEWER REPORT**

**TOPIC**

Prevalence and associated factors of low birth weight in Ethiopia: an umbrella review of a systematic review and meta-analysis

**Remark(s) 1:** This should read: “Prevalence and associated factors of low birth weight in Ethiopia: an umbrella review of systematic reviews and meta-analyses

**ABSTRACT**

**Background:**

**Lines 19-21:** Despite the reduction of neonatal morbidity and mortality, is a major goal needed to be achieved by 2030, the burden remains a major challenge for low-income countries including Ethiopia.

**Remark(s) 2:** This does not read well, rephrase.

**Lines 21-23:** Although various systematic review and meta-analysis (SRMA) studies have been conducted on LBW in Ethiopia, there is notable variation among their findings.

**Remark(s) 3:** Recast to read: “Although various systematic reviews and meta-analyses (SRMA) have been conducted on LBW in Ethiopia, there is notable variation among their findings.

**Aim: Lines 23-25:** Therefore, an umbrella review of these studies aimed to pool the inconsistent findings into a single summary estimate that could guide health policy development and planning in Ethiopia.

**Remark(s) 4:** This is inadequate…a mere summary of inconsistent findings from systematic reviews and meta-analyses does not provide a strong justification for this review. Note that this is a review of systematic reviews and meta-analyses, all of which are evidence synthesis of primary studies conducted. Typically, reviews are conducted to synthesis evidence, with the view to determining the extent, scope, and direction of existing evidence on a particular subject area which may also help to unveil gaps in literature.

**KEY WORDS**

**Remark(s) 5:** The “Umbrella review” not a MeSH Term for this topic and should be replaced with a MeSH Term.

**INTRODUCTION**

**Page 1, para 1, lines 48 & 49:** LBW is a useful public health measure of poverty, healthcare delivery, nutrition, and maternal health (2).

**Remark(s) 6:** Recast to read: “LBW is a useful public health measure of poverty, quality of healthcare delivery, quality of nutrition, and quality of maternal health (2).

**Page 1, para 1, lines 50-53:** LBW infants are more….

**Remark(s) 7:** Recast to read: “Infants with LBW are more….

**Page 1, para 2, lines 57-59:** …, low birth weight newborns are about 20 times more likely to die than with normal birth weight (10).

**Remark(s) 8:** Recast to read: “…, newborns with low birth weight are about 20 times more likely to die than with normal birth weight (10).

**Page 2, para 2, line 71:** Reduction of neonatal mortality is one of the major Sustainable Developmental Goals (SDGs) in a country like Ethiopia….

**Remark(s) 9:** Provide details on the specific SDG you are referring to.

**Page 2, para 2, lines 74-79:** Despite extensive attempts by the Ministry of Health (MoH) and non-governmental organizations (NGOs), LBW is still one of the leading causes of neonatal morbidity and mortality in Ethiopia. This may be due to limited information available about the problem. Hence, the availability of local information on the determinant factors of LBW has a major role in the management and control of the case in the country and gaining insights of such factors from this study will help the stake holders and implementers to design new as well as to strength the existing programs towards the problem.

**Remark(s) 10:** Several primary studies and systematic reviews and meta-analyses may have provided a reasonably adequate information on the subject, at least regarding the factors that account for LBW in Ethiopia. This cannot be the primary focus of this review. Rephrase this portion.

**Remark(s) 11:** “Hence, the availability of local information on the determinant factors of LBW has a major role….” Recast this to read: “Therefore, the availability of local information on the determinants of LBW has a major role….”

**Page 2, para 2, lines 79-82:** Even though, there are many systematic review and meta-analysis in Ethiopia but their finding is inconsistent, therefore, umbrella review of these studies aimed to pool the findings into a single summary estimate that can provide guidance for health policy development and planning in Ethiopia.

**Remark(s) 12:** Recast to read: “Even though, there are many systematic reviews and meta-analyses in Ethiopia, the findings are inconsistent, therefore, an umbrella review….

**Remark(s) 13:** The justification for the review is insufficient….there is need to go beyond the supposed inconsistencies in the existing reviews. The authors need to consult literature on why review of reviews are conducted. Note, you are reviewing systematic reviews and meta-analyses, which may have addressed the supposed inconsistencies found in the primary studies used.

**METHODS**

**Page 3, lines 86-89:** This method provides decision-makers with a comprehensive source of information to support clinical recommendations and guide policymakers, intervention developers, and healthcare evaluators based on prior research (26,27).

**Remark(s) 14:** This is irrelevant for the section, delete and provide a more relevant information.

**Eligibility criteria:**

**Remark(s) 15:** The authors should respond to the following:

Were all the included reviews “systematic reviews and meta-analyses”? Did you include scoping reviews? Why have the authors included studies that have not passed peer-review in this review?

**RESULTS**

**Remark(s) 16:** Provide a table to demonstrate how findings of the current review compare or differ from findings of the included systematic reviews and meta-analyses. A column in the table should show the novelty of the current review.

**DISCUSSION**

**Page 6, para 2, lines 295 & 296:** The five SRMA in this umbrella review revealed that the overall pooled prevalence of LBW in Ethiopia was 16% (95% CI 13, 18).

**Remark(s) 17:** Recast to read: “The five SRMA in this umbrella review (cite the five SRMA here) revealed that the overall pooled prevalence of LBW in Ethiopia was 16% (95% CI 13, 18).

**Page 6, para 2, lines 296-298:** The finding of this study agreed with studies conducted in Africa (49) and the global systematic analysis estimates (13). However, the result of this study was higher than those of studies conducted in SSA countries (50), and Iran (51).

**Remark(s) 18:** These sentences do not read well, recast.

**Page 6, para 3, lines 301-303:** Those infants who delivered before 37 weeks of gestation were 7.86 times more likely to have LBW compared to those delivered after 37 weeks of gestation.

**Remark(s) 19:** Recast to read: “Infants who were delivered before 37 weeks….”

**Page 6, para 3, lines 304 & 305:** This might be due to the fact that several organ of the fetus usually become mature by the end of 37 weeks of gestation.

**Remark(s) 20:** This does not read well, recast.

**Page 7, para 1, lines 307-309:** The odds of having LBW among women who did not attend ANC follow-up were 2.4 times higher than who did attend ANC.

**Remark(s) 21:** Introduce the word “those” in between the words “than” and “who”.

**Page 7, para 2, line 317:** In this study, women with PIH had 4.20 times higher odds of giving LBW compared to normotensive women.

**Remark(s) 22:** Recast to read: “…higher odds of giving birth to babies with LBW….” Or “…higher odds of delivering babies with LBW….” Apply this throughout the manuscript.

**Page 7, para 2, lines 317-321:** In this study, women with PIH had 4.20 times higher odds of giving LBW compared to normotensive women. This finding is in line with the WHO secondary analysis survey conducted in LMICs (56), Indonesia (52), and Malaysia (54). This might be due to endothelial cell injury and vasoconstriction of blood vessels, resulting in reduced utero-placental blood perfusion leading to LBW (57).

**Remark(s) 23:** There is a problem with this paragraph. “This might be due to endothelial cell injury and vasoconstriction of blood….” Are the authors seeking to explain their finding or those of the WHO secondary surveys (52,54,56)? This can leave the audiences confused and must be corrected.

Rather, the point should read: “In this study, women with PIH had 4.20 times higher odds of giving LBW compared to normotensive women. This might be due to endothelial cell injury and vasoconstriction of blood vessels, resulting in reduced utero-placental blood perfusion leading to LBW (57). This finding is in line with the WHO secondary analysis survey conducted in LMICs (56), Indonesia (52), and Malaysia (54). There are similar errors of construction throughout the discussion which must be corrected to guarantee clarity.

**Page 8, para 1, lines 331-331:** Women who gave birth before 24 months had 2.96 times higher odds of having LBW compared to those with birth interval greater than 24 months.

**Remark(s) 24:** Recast to read: “…had 2.96 times higher odds of delivering babies with LBW compared….” Correct this throughout the manuscript.

**LIMITATION OF THE REVIEW**

**Remark(s) 25:** The section is woefully inadequate; there are more issues that must be reported. For instance, inclusion of unpublished reviews raises concerns over the reliability of the study.

**CONCLUSION**

**Remark(s) 26:** The section is inadequate; provide a more comprehensive conclusion to cover all the key findings and also add recommendations. Note that the recommendations must be directed at specific organizations and individuals for action. Additionally, you must suggest possible areas for future research direction in the area.

**COMMENTS FOR THE AUTHORS**

Overall, the authors have made a good effort at dealing with a subject of high public health importance. However, the paper will need substantial revision to make it suitable for publication. Apart from the remarks provided above, the authors must conduct a thorough and rigorous English Language editing of the paper.
