# Supplementary material for: Prevalence of low birth weight and associated factors in Ethiopia: An umbrella review of systematic review and meta-analyses
Source: PLOS Glob Public Health. 2025 May 8;5(5):e0004556. doi: 10.1371/journal.pgph.0004556 (PMC12061095; doi:10.1371/journal.pgph.0004556)
Supplement: Attachment — Submitted filename: REVIEWER.docx [file pgph.0004556.s010.docx]

REVIEWER’S COMMENTS

**Line 64:** Instead of providing the abbreviation for ANC, which stands for antenatal care, you provided the abbreviation in line 228. The abbreviation should start at line 64.

**Line 64:** You did not use an abbreviation for the word 'preterm birth' in this line, but you did use one in line 301. Since the word first appeared in line 64, use the abbreviation there and not on line 301.

**Line 78:** It is stakeholders and not stake holders.

**Line 78:** Replace "implementers" with "policymakers". Implementers are the civil servants who implement policies initiated by policymakers.

**Line 80:** The right phrase should be ‘their findings are’ and not ‘their finding is.’

**Line 85:** Among the types of reviews, these are the most common: systematic review, narrative review, meta-analysis, scoping review, literature review, and rapid review. Since your review is an umbrella review, can you give a brief definition of it?

**Line 102:** Use only the abbreviation SRMA in line 102

**Line 163:** Characteristics of included systematic review and meta-analysis studies. Columns 8 and 9 contain information that not everyone can understand. For instance, what is JIB? If JIB is a critical appraisal tool, inform your readers that it is.

**Line 172:** Please remove both parentheses from the abbreviation (LBW).

**Line 182:** This line should be “Table 1 presents the general characteristics of the included systematic reviews and meta-analysis studies.’

**Line 186:** All titles in Table 1 should be uniform. Column 5 title is not.

**Line 190:** it would be beneficial for the authors to explain what is meant by a *primary study* for clarity, especially for readers who may not be familiar with research terminology. A brief explanation would help clarify that *primary studies* are the original research studies providing firsthand data, distinct from systematic reviews or meta-analyses that synthesize findings from multiple primary sources. This additional clarity is particularly useful in fields where readers may come from various backgrounds and may not be accustomed to the distinctions between primary, secondary, and tertiary sources in research literature. A short, clear definition within the methodology or background section would make the text more accessible and help readers follow the mapping of evidence sources across reviews.

**Line 228:** Use only the abbreviation ANC.

**Line 304:** Replace "several organ" with "several organs."

Finally, it is inappropriate to use ‘don’t’ in a research paper for publication. This is informal and not suitable. Your manuscript contains several don'ts. Please replace all with do not.
