# Supplementary material for: Prevalence of low birth weight and associated factors in Ethiopia: An umbrella review of systematic review and meta-analyses
Source: PLOS Glob Public Health. 2025 May 8;5(5):e0004556. doi: 10.1371/journal.pgph.0004556 (PMC12061095; doi:10.1371/journal.pgph.0004556)
Supplement: Attachment — Submitted filename: response_to_reviewer_auresp_3.docx [file pgph.0004556.s011.docx]

**Prevalence and associated factors of low birth weight in Ethiopia: an umbrella review of systematic review and meta-analysis:**

The authors thank the editor and reviewers for giving us a further opportunity to update our manuscript.

Editors: The authors addressed all given comments by reviewer point by points.

**Reviewer # 3: TOPIC**

Prevalence and associated factors of low birth weight in Ethiopia: an umbrella review of a systematic review and meta-analysis

Remark(s) 1: This should read: “Prevalence and associated factors of low birth weight in Ethiopia: an umbrella review of systematic reviews and meta-analyses

**Authors’ response:** Thank you! The authors made revision

ABSTRACT
Background:
Lines 19-21: Despite the reduction of neonatal morbidity and mortality, is a major goal needed to be achieved by 2030, the burden remains a major challenge for low-income countries including Ethiopia.

Remark(s) 2: This does not read well, rephrase.

**Authors’ response:** Thank you! The authors made revision

Lines 21-23: Although various systematic review and meta-analysis (SRMA) studies have been conducted on LBW in Ethiopia, there is notable variation among their findings.
Remark(s) 3: Recast to read: “Although various systematic reviews and meta-analyses (SRMA) have been conducted on LBW in Ethiopia, there is notable variation among their findings.

**Authors’ response**: Thank you! The authors made changes.

Aim: Lines 23-25: Therefore, an umbrella review of these studies aimed to pool the inconsistent findings into a single summary estimate that could guide health policy development and planning in Ethiopia.

Remark(s) 4: This is inadequate…a mere summary of inconsistent findings from systematic reviews and meta-analyses does not provide a strong justification for this review. Note that this is a review of systematic reviews and meta-analyses, all of which are evidence synthesis of primary studies conducted. Typically, reviews are conducted to synthesis evidence, with the view to determining the extent, scope, and direction of existing evidence on a particular subject area which may also help to unveil gaps in literature.

**Authors’ response:** Thank you! The authors made revision

KEY WORDS

Remark(s) 5: The “Umbrella review” not a MeSH Term for this topic and should be replaced with a MeSH Term.

**Authors’ response:** Thank you! The authors made revision

INTRODUCTION
Page 1, para 1, lines 48 & 49: LBW is a useful public health measure of poverty, healthcare delivery, nutrition, and maternal health (2).

Remark(s) 6: Recast to read: “LBW is a useful public health measure of poverty, quality of healthcare delivery, quality of nutrition, and quality of maternal health (2).

**Authors’ response:** Thank you! The authors made revision

Page 1, para 1, lines 50-53: LBW infants are more….

Remark(s) 7: Recast to read: “Infants with LBW are more….

**Authors’ response:** Thank you! The authors made revision

Page 1, para 2, lines 57-59: …, low birth weight newborns are about 20 times more likely to die

than with normal birth weight (10).

Remark(s) 8: Recast to read: “…, newborns with low birth weight are about 20 times more likely to die than with normal birth weight (10).

**Authors’ response:** Thank you! The authors made revision

Page 2, para 2, line 71: Reduction of neonatal mortality is one of the major Sustainable Developmental Goals (SDGs) in a country like Ethiopia….

Remark(s) 9: Provide details on the specific SDG you are referring to.

**Authors’ response:** Thank you! The authors made revision

Page 2, para 2, lines 74-79: Despite extensive attempts by the Ministry of Health (MoH) and non-governmental organizations (NGOs), LBW is still one of the leading causes of neonatal morbidity and mortality in Ethiopia. This may be due to limited information available about the problem. Hence, the availability of local information on the determinant factors of LBW has a major role in the management and control of the case in the country and gaining insights of such factors from this study will help the stake holders and implementers to design new as well as to strength the existing programs towards the problem.

Remark(s) 10: Several primary studies and systematic reviews and meta-analyses may have provided a reasonably adequate information on the subject, at least regarding the factors that account for LBW in Ethiopia. This cannot be the primary focus of this review. Rephrase this portion.

Remark(s) 11: “Hence, the availability of local information on the determinant factors of LBW has a major role….” Recast this to read: “Therefore, the availability of local information on the determinants of LBW has a major role….”

Page 2, para 2, lines 79-82: Even though, there are many systematic review and meta-analysis in Ethiopia but their finding is inconsistent, therefore, umbrella review of these studies aimed to pool the findings into a single summary estimate that can provide guidance for health policy development and planning in Ethiopia.

Remark(s) 12: Recast to read: “Even though, there are many systematic reviews and meta-analyses in Ethiopia, the findings are inconsistent, therefore, an umbrella review….
Remark(s) 13: The justification for the review is insufficient….there is need to go beyond the supposed inconsistencies in the existing reviews. The authors need to consult literature on why review of reviews are conducted. Note, you are reviewing systematic reviews and meta-analyses, which may have addressed the supposed inconsistencies found in the primary studies used.

**Authors’ response:** Thank you! The authors made revision

METHODS
Page 3, lines 86-89: This method provides decision-makers with a comprehensive source of information to support clinical recommendations and guide policymakers, intervention developers, and healthcare evaluators based on prior research (26,27).
Remark(s) 14: This is irrelevant for the section, delete and provide a more relevant information.

**Authors’ response:** Thank you! The authors made changes

Eligibility criteria:

Remark(s) 15: The authors should respond to the following:

Were all the included reviews “systematic reviews and meta-analyses”?

**Authors’ response:** Yes, all included in the reviews were SRMA studies.

Did you include scoping reviews?

**Authors’ response:** No

Why have the authors included studies that have not passed peer-review in this review?

**Authors’ response:** Thank you! Authors seek to capture a broader range of data by including unpublished studies, which can provide a more comprehensive view of the available evidence and minimize bias. Reference: Aromataris E, Munn Z (Editors) The Joanna Briggs Institute,. Joanna Briggs Institute Reviewer's Manual 2017. Available from http://reviewers manual.joannabriggs.org/.

RESULTS

Remark(s) 16: Provide a table to demonstrate how findings of the current review compare or differ from findings of the included systematic reviews and meta-analyses. A column in the table should show the novelty of the current review.

**Authors’ response:** Thank you! The table below compares the findings of the included systematic reviews and meta-analyses with those of the current review. The right-hand column (Remarks) highlights the novelty of the current review.

| Authors of SRMA | SRMA results | Umbrella results | Remark (novelty of the current findings) |
| --- | --- | --- | --- |
| Habtegiorgis et al., (22) | maternal age, Ante Natal Care follow up, Gestational Age, Birth Interval, Pregnancy Induced Hypertension, and maternal anemic status | Not attending ANC follow-up, Gestational age <37weeks, Pregnancy-induced hypertension(PIH), Rural residency, Pregnancy interval <24 months, Maternal age<20, Iron/ folic acid supplementation(IFAS) | Rural residence and IFAS is novel finding of this review |
| Endalamaw et al.,2018 (39) | Maternal age < 20 years, pregnancy interval < 24 months, BMI < 18.5 kg/m2, and gestational age < 37 weeks at birth |  | Not attending ANC follow-up, PIH, IFAS, rural residence is a novel findings of this review |
| Katiso et al., 2020 (41) | female babies, prematurity, not attending prenatal care, pregnancy-induced hypertension, and rural areas |  | Pregnancy interval <24 months, IFAS, Maternal age<20 is the novel findings of this review |
| Tamirat et al.,2020 (42) | Gestational age < 37weeks, no antenatal care, rural residence and women with medical illness during pregnancy |  | Pregnancy interval <24 months, IFAS, Maternal age<20, PIH is the novel findings of this review |
| Getaneh et al., 2020 (43) | PIH |  | Not attending ANC follow-up, GA <37wks, Rural residency, Pregnancy interval <24 months, Maternal age<20, Iron/ folic acid supplementation(IFAS) is the novel findings of this review |
| Zenebe et al., 2021 (44) and Tegegne et al.,2021 (45) | IFAS |  | Not attending ANC follow-up, GA <37wks, Rural residency, Pregnancy interval <24 months, Maternal age<20 is the novel findings of this review |

DISCUSSION
Page 6, para 2, lines 295 & 296: The five SRMA in this umbrella review revealed that the overall

pooled prevalence of LBW in Ethiopia was 16% (95% CI 13, 18).

Remark(s) 17: Recast to read: “The five SRMA in this umbrella review (cite the five SRMA here) revealed that the overall pooled prevalence of LBW in Ethiopia was 16% (95% CI 13, 18).

**Authors’ response:** Thank you! The authors made revision

Page 6, para 2, lines 296-298: The finding of this study agreed with studies conducted in Africa (49) and the global systematic analysis estimates (13). However, the result of this study was higher than those of studies conducted in SSA countries (50), and Iran (51).

Remark(s) 18: These sentences do not read well, recast.

**Authors’ response:** Thank you! The authors made revision

Page 6, para 3, lines 301-303: Those infants who delivered before 37 weeks of gestation were 7.86 times more likely to have LBW compared to those delivered after 37 weeks of gestation.
Remark(s) 19: Recast to read: “Infants who were delivered before 37 weeks….”

**Authors’ response:** Thank you! The authors made revision

Page 6, para 3, lines 304 & 305: This might be due to the fact that several organ of the fetus usually become mature by the end of 37 weeks of gestation.

Remark(s) 20: This does not read well, recast.

**Authors’ response:** Thank you! The authors made revision

Page 7, para 1, lines 307-309: The odds of having LBW among women who did not attend ANC follow-up were 2.4 times higher than who did attend ANC.

Remark(s) 21: Introduce the word “those” in between the words “than” and “who”.

**Authors’ response:** Thank you! The authors made revision

Page 7, para 2, line 317: In this study, women with PIH had 4.20 times higher odds of giving LBW compared to normotensive women.

Remark(s) 22: Recast to read: “…higher odds of giving birth to babies with LBW….” Or “…higher odds of delivering babies with LBW….” Apply this throughout the manuscript.

**Authors’ response:** Thank you! The authors made revision

Page 7, para 2, lines 317-321: In this study, women with PIH had 4.20 times higher odds of giving LBW compared to normotensive women. This finding is in line with the WHO secondary analysis survey conducted in LMICs (56), Indonesia (52), and Malaysia (54). This might be due to endothelial cell injury and vasoconstriction of blood vessels, resulting in reduced utero-placental blood perfusion leading to LBW (57).

Remark(s) 23: There is a problem with this paragraph. “This might be due to endothelial cell injury and vasoconstriction of blood….” Are the authors seeking to explain their finding or those of the WHO secondary surveys (52,54,56)? This can leave the audiences confused and must be corrected.

Rather, the point should read: “In this study, women with PIH had 4.20 times higher odds of giving LBW compared to normotensive women. This might be due to endothelial cell injury and vasoconstriction of blood vessels, resulting in reduced utero-placental blood perfusion leading to LBW (57). This finding is in line with the WHO secondary analysis survey conducted in LMICs (56), Indonesia (52), and Malaysia (54). There are similar errors of construction throughout the discussion which must be corrected to guarantee clarity.

**Authors’ response:** Thank you! The authors made revision

Page 8, para 1, lines 331-331: Women who gave birth before 24 months had 2.96 times higher odds of having LBW compared to those with birth interval greater than 24 months.
Remark(s) 24: Recast to read: “…had 2.96 times higher odds of delivering babies with LBW compared….” Correct this throughout the manuscript.

**Authors’ response:** Thank you! The authors made revision

LIMITATION OF THE REVIEW

Remark(s) 25: The section is woefully inadequate; there are more issues that must be reported. For instance, inclusion of unpublished reviews raises concerns over the reliability of the study.

**Authors’ response:** Thank you! The authors made revision

CONCLUSION
Remark(s) 26: The section is inadequate; provide a more comprehensive conclusion to cover all the key findings and also add recommendations. Note that the recommendations must be directed at specific organizations and individuals for action. Additionally, you must suggest possible areas for future research direction in the area.

**Authors’ response:** Thank you! The authors made revision

**REVIEWER’S #5 COMMENTS**

Line 64: Instead of providing the abbreviation for ANC, which stands for antenatal care, you

provided the abbreviation in line 228. The abbreviation should start at line 64.

Line 64: You did not use an abbreviation for the word &#39;preterm birth&#39; in this line, but you did use one in line 301. Since the word first appeared in line 64, use the abbreviation there and not on line 301.

**Authors’ response:** Thank you! The authors made revision

Line 78: It is stakeholders and not stake holders.

Line 78: Replace &quot;implementers&quot; with &quot;policymakers&quot;. Implementers are the civil servants who implement policies initiated by policymakers.

**Authors’ response:** Thank you! The authors made revision

Line 80: The right phrase should be ‘their findings are’ and not ‘their finding is.’

**Authors’ response:** Thank you! The authors made revision

Line 85: Among the types of reviews, these are the most common: systematic review, narrative review, meta-analysis, scoping review, literature review, and rapid review. Since your review is an umbrella review, can you give a brief definition of it?

**Authors’ response:** Thank you! The authors made revision

Line 102: Use only the abbreviation SRMA in line 102

**Authors’ response:** Thank you! The authors made revision

Line 163: Characteristics of included systematic review and meta-analysis studies. Columns

8 and 9 contain information that not everyone can understand. For instance, what is JIB? If

JIB is a critical appraisal tool, inform your readers that it is.

**Authors’ response:** Thank you! The authors made revision

Line 172: Please remove both parentheses from the abbreviation (LBW).

**Authors’ response:** Thank you! The authors made revision

Line 182: This line should be “Table 1 presents the general characteristics of the included

systematic reviews and meta-analysis studies.’

**Authors’ response:** Thank you! The authors made revision

Line 186: All titles in Table 1 should be uniform. Column 5 title is not.

**Authors’ response:** Thank you! The authors made revision

Line 190: it would be beneficial for the authors to explain what is meant by a *primary study*

for clarity, especially for readers who may not be familiar with research terminology. A brief explanation would help clarify that *primary studies* are the original research studies providing firsthand data, distinct from systematic reviews or meta-analyses that synthesize findings from multiple primary sources. This additional clarity is particularly useful in fields where readers may come from various backgrounds and may not be accustomed to the distinctions between primary, secondary, and tertiary sources in research literature. A short, clear definition within the methodology or background section would make the text more accessible and help readers follow the mapping of evidence sources across reviews.

**Authors’ response:** Thank you! The authors made revision

Line 228: Use only the abbreviation ANC.

**Authors’ response:** Thank you! The authors made revision

Line 304: Replace &quot;several organ&quot; with &quot;several organs.&quot;

**Authors’ response:** Thank you! The authors made revision

Finally, it is inappropriate to use ‘don’t’ in a research paper for publication. This is informal

and not suitable. Your manuscript contains several don&#39;ts. Please replace all with do not.

**Authors’ response:** Thank you! The authors made revision
