# Supplementary material for: Prevalence of low birth weight and associated factors in Ethiopia: An umbrella review of systematic review and meta-analyses
Source: PLOS Glob Public Health. 2025 May 8;5(5):e0004556. doi: 10.1371/journal.pgph.0004556 (PMC12061095; doi:10.1371/journal.pgph.0004556)
Supplement: Attachment — Submitted filename: Comments.docx [file pgph.0004556.s012.docx]

**Comments**

Line 19: There should be **hyphens** in “low- and middle-income countries” Also in Line 70

Line 64: A comma is need after “in 2020”

Line 77: Please use only the abbreviation “LBW”

Line 112: You have **three parentheses (((** before “infant” instead of one parenthesis

Line 305-307: The sentence written is not correct. This is the sentence: “Infants who were delivered before 37 weeks of gestation were 7.86 times more likely to delivering babies with LBW compared to those delivered after 37 weeks of gestation.”

**This should be the correct sentence:**

"Infants who were delivered before 37 weeks of gestation were 7.86 times more likely to have a LBW compared to those delivered after 37 weeks of gestation."

**In the conclusion**, use only the abbreviations of LWB, PIH, IFAS, and MoH. You have given the full meaning of these abbreviations in previous sections, so repeating them again in the conclusion is not ideal.
