# Supplementary material for: Prevalence of low birth weight and associated factors in Ethiopia: An umbrella review of systematic review and meta-analyses
Source: PLOS Glob Public Health. 2025 May 8;5(5):e0004556. doi: 10.1371/journal.pgph.0004556 (PMC12061095; doi:10.1371/journal.pgph.0004556)
Supplement: Attachment — Submitted filename: response_to_reviewer_auresp_4.docx [file pgph.0004556.s013.docx]

**Prevalence and associated factors of low birth weight in Ethiopia: an umbrella review of systematic review and meta-analysis:**

The authors thank the editor and reviewers for giving us a further opportunity to update our manuscript.

Editors: The authors addressed all given comments by reviewer point by points.

**Reviewer # 3:**

The authors have done well in responding to the comments but not as expected. The justification for the review is weak and needs further revision, the table on Remark(s) 16: Provide a table to demonstrate how findings of the current review compare or differ from findings of the included systematic reviews and meta-analyses. A column in the table should show the novelty of the current review, was not adequately done. In fact, the table provided by the authors is not clear and must be revised with further details.

**Authors’ response:** Thank you dear reviewer! As you know, this umbrella review finding is to consolidate inconsistent findings reported in different systematic reviews and meta-analyses into a single summary estimate to provide a robust synthesis of evidence. The table below compares the findings of the included systematic reviews and meta-analyses with those of the current review. The right-hand column (Remarks) highlights the robust result of the current review. For instance, findings from Habtegiorgis et al., (22) one of systematic reviews and meta-analyses study included in these umbrella review indicated that maternal age, antenatal care, gestational age, birth interval, pregnancy-induced hypertension, and maternal anemia are associated with low birth weight. Additionally, this umbrella review identified other factors, such as rural residence and iron/folic acid supplementation, derived from the systematic reviews and meta-analyses included in this umbrella review.

| Authors of SRMA | SRMA results | Umbrella results | Remark (novelty of the current findings) |
| --- | --- | --- | --- |
| Habtegiorgis et al., (22) | maternal age, Ante Natal Care follow up, Gestational Age, Birth Interval, Pregnancy Induced Hypertension, and maternal anemic status | Not attending ANC follow-up, Gestational age <37weeks, Pregnancy-induced hypertension(PIH), Rural residency, Pregnancy interval <24 months, Maternal age<20, Iron/ folic acid supplementation(IFAS) | Rural residence and IFAS is novel finding of this review |
| Endalamaw et al.,2018 (39) | Maternal age < 20 years, pregnancy interval < 24 months, BMI < 18.5 kg/m2, and gestational age < 37 weeks at birth |  | Not attending ANC follow-up, PIH, IFAS, rural residence is a novel findings of this review |
| Katiso et al., 2020 (41) | female babies, prematurity, not attending prenatal care, pregnancy-induced hypertension, and rural areas |  | Pregnancy interval <24 months, IFAS, Maternal age<20 is the novel findings of this review |
| Tamirat et al.,2020 (42) | Gestational age < 37weeks, no antenatal care, rural residence and women with medical illness during pregnancy |  | Pregnancy interval <24 months, IFAS, Maternal age<20, PIH is the novel findings of this review |
| Getaneh et al., 2020 (43) | PIH |  | Not attending ANC follow-up, GA <37wks, Rural residency, Pregnancy interval <24 months, Maternal age<20, Iron/ folic acid supplementation(IFAS) is the novel findings of this review |
| Zenebe et al., 2021 (44) and Tegegne et al.,2021 (45) | IFAS |  | Not attending ANC follow-up, GA <37wks, Rural residency, Pregnancy interval <24 months, Maternal age<20 is the novel findings of this review |

**REVIEWER’S #5 COMMENTS**

Line 19: There should be hyphens in “low- and middle-income countries” Also in Line 70

**Authors’ response:** Thank you! The authors made revision

Line 64: A comma is need after “in 2020”

**Authors’ response:** The authors made revision

Line 77: Please use only the abbreviation “LBW”

**Authors’ response:** The authors made revision

Line 112: You have three parentheses **(((**before “infant” instead of one parenthesis

**Authors’ response:** The authors made revision

Line 305-307: The sentence written is not correct. This is the sentence: “Infants who were delivered before 37 weeks of gestation were 7.86 times more likely to delivering babies with LBW compared to those delivered after 37 weeks of gestation.”

**Authors’ response:** Thank you! The authors accepted comments and made revision.

In the conclusion, use only the abbreviations of LWB, PIH, IFAS, and MoH. You have given the full meaning of these abbreviations in previous sections, so repeating them again in the conclusion is not ideal.

**Authors’ response:** Thank you! The authors made revision
